# Rapid Screening of Novel Agents for Combination Therapy in Sarcomas

**DOI:** 10.1155/2013/365723

**Published:** 2013-10-24

**Authors:** Christopher L. Cubitt, Jiliana Menth, Jana Dawson, Gary V. Martinez, Parastou Foroutan, David L. Morse, Marilyn M. Bui, G. Douglas Letson, Daniel M. Sullivan, Damon R. Reed

**Affiliations:** ^1^Chemical Biology and Molecular Medicine, H. Lee Moffitt Cancer Center and Research Institute, 12902 Magnolia Drive, Tampa, FL 33612, USA; ^2^Translational Research Lab, H. Lee Moffitt Cancer Center and Research Institute, 12902 Magnolia Drive, Tampa, FL 33612, USA; ^3^Small Animal Imaging Lab, H. Lee Moffitt Cancer Center and Research Institute, 12902 Magnolia Drive, Tampa, FL 33612, USA; ^4^Sarcoma Program, H. Lee Moffitt Cancer Center and Research Institute, 12902 Magnolia Drive, Tampa, FL 33612, USA; ^5^Anatomic Pathology Department, H. Lee Moffitt Cancer Center and Research Institute, 12902 Magnolia Drive, Tampa, FL 33612, USA

## Abstract

For patients with sarcoma, metastatic disease remains very difficult to cure, and outcomes remain less than optimal. Treatment options have not largely changed, although some promising gains have been made with single agents in specific subtypes with the use of targeted agents. Here, we developed a system to investigate synergy of combinations of targeted and cytotoxic agents in a panel of sarcoma cell lines. Agents were investigated alone and in combination with varying dose ratios. Dose-response curves were analyzed for synergy using methods derived from Chou and Talalay (1984). A promising combination, dasatinib and triciribine, was explored in a murine model using the A673 cell line, and tumors were evaluated by MRI and histology for therapy effect. We found that histone deacetylase inhibitors were synergistic with etoposide, dasatinib, and Akt inhibitors across cell lines. Sorafenib and topotecan demonstrated a mixed response. Our systematic drug screening method allowed us to screen a large number of combinations of sarcoma agents. This method can be easily modified to accommodate other cell line models, and confirmatory assays, such as animal experiments, can provide excellent preclinical data to inform clinical trials for these rare malignancies.

## 1. Introduction

Sarcomas account for 10% of pediatric diagnoses, 8% of cancers in the adolescent/young adult population, and 1% of adult cancers [1]. This diverse group of malignancies is often lethal in surgically unresectable, recurrent, or metastatic settings. Different subtypes predominate in different age groups, with rhabdomyosarcoma, osteosarcoma, and Ewing sarcoma predominating in children and young adults and leiomyosarcoma, liposarcoma, and other soft tissue sarcomas predominating in older adults. Chemotherapy has demonstrated clinical benefit in patients with advanced disease; however, for patients with advanced metastatic soft tissue sarcoma, the prognosis remains poor, with disease-free survival of 5 years or less than 25%; therefore, novel therapeutic strategies are needed.

Targeted therapy has shown promise in subtypes of sarcoma, with c-Kit mutant gastrointestinal stromal tumor demonstrating the most clinical efficacy to date [2]. Signaling pathways have long been known to be active in sarcomas, with Src being the first discovered oncogene. The success of single-agent targeted therapy in gastrointestinal stromal tumors has not been reproduced in other sarcomas, although investigations regarding targeted therapies with clinical benefit, particularly in combination, continue. Single agents or combinations that have demonstrated preclinical activity in sarcoma models are a rational choice for further clinical investigations. However, single agents tested at various dose levels over the years have shown a modest impact for relapsed and refractory sarcomas. In fact, the current clinical benchmark for activity in the second-line setting is a 40% 3-month progression-free survival [3]. This mark, demonstrating at least a promise for targeted therapies in patients with soft tissue sarcomas, was met by the targeted agent sunitinib in a phase II study at the Moffitt Cancer Center [4]. Other promising sarcoma agents include histone deacetylase (HDAC) inhibitors, tyrosine kinase inhibitors, and topoisomerase inhibitors.

Despite the promise shown by single-agent activity *in vitro*, clinical investigations have demonstrated that combinations of chemotherapy are often required to reliably induce responses and improve survival in sarcomas. Pediatric malignancies have traditionally seen improved response and cure rates with combinations of chemotherapies, with current standards of care employing 2–7 agents in the front-line setting for solid tumors. To this end, in this study, we explored combinations of cytotoxic and targeted agents in multiple sarcoma cell lines, including rhabdomyosarcoma, osteosarcoma, and Ewing sarcoma. In particular, we focused on topoisomerase inhibitors in combination with targeted agents, observing synergy across sarcoma cell lines in combination with selected tyrosine kinase inhibitors and HDAC inhibitors. We sought to establish a platform to allow for rapid determination of drug combination effects on tumor cell death and to assess for synergy, additivity, or antagonism across multiple sarcoma histologies.

## 2. Materials and Methods

### 2.1. Investigational Agents

Agents used included both cytotoxic and targeted agents. Many of these agents were obtained through the National Cancer Institute's Cancer Therapy Evaluation Program (see Table 1). Structures for all agents are publicly available. Combinations of investigational agents were all performed under material transfer agreements. All possible combinations of agents that were allowable were tested. Requests focused on tyrosine kinase inhibitors, HDAC inhibitors, though other agents with rationale were considered on a case by case basis.

### 2.2. Cell Culture

Sarcoma cell lines were obtained from the ATCC (Manassas, VA). Cells were maintained in RPMI or DMEM with 10% fetal bovine serum according to manufacturer recommendations. Cells were grown at 37°C and 5% CO_2_. All cell lines tested free of mycoplasma every 3 months with MycoAlert tests (Lonza Rockland, Inc., Rockland, ME). Cell line identity was confirmed using StemElite ID system (Promega Corp., Madison, WI) using the manufacturer's instructions and the ATCC STR profile database.

### 2.3. Cell Viability Assays

The activity levels of drugs alone and in combination were determined by a high-throughput CellTiter-Blue (Promega Corp.) cell viability assay. Cells (1.2–2 × 10^3^) were plated in each well of 384-well plates using a Precision XS liquid handling station (Bio-Tek Instruments, Inc., Winooski, VT) and incubated overnight. A liquid handling station was used to serially dilute all drugs in media, and 5 *μ*L was added to four replicate wells and an additional four control wells received a diluent control without drug. At the end of the incubation period with drugs, 5 *μ*L of CellTiter-Blue reagent was added to each well. The fluorescence of the product of viable cells' bioreduction, resorufin (579 nm excitation/584 nm emission), was measured with a Synergy 4 microplate reader (Bio-Tek Instruments, Inc.). The fluorescence data were transferred to Microsoft Excel to calculate the percent viability. We determined IC50 values using a sigmoidal equilibrium model regression and XLfit version 5.2 (ID Business Solutions Ltd.). The IC50 values obtained from single-drug cell viability assays were used to design subsequent drug combination experiments.

### 2.4. Analysis of Additive and Synergistic Effects

For drug combination experiments, the cell viability assays were performed as described above, and the results were analyzed for synergistic, additive, or antagonistic effects using the combination index (CI) method developed by Chou and Talalay [5]. For the application of this method, the drug concentration dilutions were used at fixed dose molar ratios based on the IC50 levels of each drug obtained from preliminary experiments (e.g., 50 : 1, 2 : 5, and 1 : 250). Briefly, the dose-effect curve for each drug alone was determined based on experimental observations using the median-effect principle and then compared to the effect achieved with a combination of the two drugs to derive a CI value. This method involves plotting dose-effect curves, for each agent and their combination, using the median-effect equation: *f*_*a*_/*f*_*u*_ = (*D*/*Dm*)*m*, where *D* is the dose of the drug, *Dm* is the dose required for a 50% effect (equivalent to IC50), *f*_*a*_ and *f*_*u*_ are the affected and unaffected fractions, respectively (*f*_*a*_ = 1 − *f*_*u*_), and *m* is the exponent signifying the sigmoidicity of the dose-effect curve. XLfit software was used to calculate the values of *Dm* and *m*. The CI used for the analysis of the drug combinations was determined by the isobologram equation for mutually nonexclusive drugs that have different modes of action: CI = (*D*)_1_/(*Dx*)_1_ + (*D*)_2_/(*Dx*)_2_ + (*D*)_1_(*D*)_2_/(*Dx*)_1_(*Dx*)_2_, where (*Dx*)_1_ and (*Dx*)_2_ in the denominators are the doses (or concentrations) for *D1* (drug 1) and *D2* (drug 2) alone that gives *x*% inhibition, whereas (*D*)_1_ and (*D*)_2_ in the numerators are the doses of drug 1 and drug 2 in combination that also inhibited *x*% (i.e., isoeffective). CI < 1, CI = 1, and CI > 1 indicate synergism, additive effects, and antagonism, respectively. A confidence interval of <0.1 is represented as +++++ and indicates strong synergism by this method. Other CI symbols and description of effect of combinations are as follows: 0.1–0.3, ++++, strong synergism; 0.3–0.7, +++, synergism; 0.7–0.85, ++, moderate synergism; 0.85–0.90, +, slight synergism; 0.90–1.10, ±, nearly additive; 1.10–1.20, −, slight antagonism; 1.20–1.45, −−, moderate antagonism; 1.45–3.3, −−−, antagonism; 3.3–10, −−−−, strong antagonism; >10, −−−−−, very strong antagonism.

Excess over the highest single agent (EOHSA) was calculated using MATLAB scripts provided by Brian Roberts of Merck & Co. For this method, the fraction unaffected was first calculated from dose-response data using a Michaelis-Menten model with Hill-type kinetics and incomplete inhibition. Specifically, EOHSA is calculated from the area between the measured combination and HSA response surfaces, where the “highest single agent” (HSA) is simply the higher of two single-agent effects at corresponding concentrations. The scripts were used to regress a best-fit CI value to a set of inhibitions yielded by two inhibitors, and predicted dose-response curves for a given CI were generated. Lastly, the area and a *P* value between the curves for actual data and HSA-predicted curves were calculated and averaged across replicate experiments.

### 2.5. Apoptosis Assay

Caspase 3/7 activation was measured using a 384-well plate based Caspase-Glo 3/7 (Promega) luminescent assay. Cells were treated for 24 hours with serial dilutions of each compound or a combination of two drugs.

### 2.6. Mouse Xenograft with A673 Ewing Sarcoma Cell Line

Animal experiments were carried out in strict accordance with recommendations in the Guide for the Care and Use of Laboratory Animals of the National Institutes of Health. The protocol was approved by the University of South Florida Institutional Animal Care and Use Committee (Application 2805).

Twenty-four, 3-month-old, male Balb/c Nu/Nu mice were injected subcutaneously with 10^6^ cells on the right flank. The cells were mixed in a solution consisting of 50 *μ*L PBS and 50 *μ*L matrigel. Mice were separated into 4 groups: a control group, two groups receiving either dasatinib or triciribine, and a group receiving a combination of dasatinib and triciribine. Treated mice received dasatinib at 200 mg/kg daily, administered orally in a citrate solution, and/or triciribine at 2 mg/kg daily, given by intraperitoneal injection in a 40% DMSO solution with PBS equaling 100 *μ*L. Both agents were given every 24 hours from Monday through Friday, every week starting one week after cells were injected. All mice were weighed daily, to the milligram. Caliper measurements in two directions of the tumors were taken daily to observe the growth. The tumors were allowed to grow to a diameter of 1.5–2.0 cm in either direction, with magnetic resonance imaging (MRI) performed at regular intervals, after which animals were sacrificed.

The tumor tissue was formalin fixed and paraffin embedded. Tumor was sectioned to 4 *μ*m thick and stained with hematoxylin and eosin (H&E). We evaluated the therapy effect by examination under light microscopy; observed results were semiquantitatively analyzed by measuring percentage of viable tumor cells, necrosis, and fibrosis. The pathologist was blinded to the treatment.

### 2.7. MRI Methods

Mice were anesthetized with 1% isoflurane in O_2_ and placed into a SWIFT insertion tube cradle fitted with a pressure-sensitive respiration pad beneath the animal. Body temperature was monitored with a fiber-optic rectal temperature probe and maintained at 37°C while being controlled using a small animal monitoring system (SA Instruments, Stony Brook, NY). All imaging was carried out at 7 Tesla using a horizontal bore Agilent ASR 310 MRI instrument (Agilent Technologies, Santa Clara, CA) equipped with actively shielded gradients capable of 400 mT/m gradient strength. Using a 35 mm inner diameter Litzcage coil (Doty Scientific, Inc.), we obtained T_2_-weighted fast spin-echo images in axial planes that spanned the volume of the tumor. Imaging parameters for these images were repetition time (TR) = 2400 ms, elective echo time (TE_eff_) = 72, echo spacing = 18 ms, echo train length (ETL) = 8, a field of view of 40 × 40 mm^2^, a matrix size of 128 × 128, 15 slices, a slice thickness of 1.25 mm, and an acquisition bandwidth of 100 kHz. To achieve fat suppression for each TR period, a 10 ms duration Gaussian saturation pulse was applied 1004 Hz upfield from water with a flip angle of 90°.

The data were analyzed using in-house scripts coded in Matlab (Mathworks, Inc., Natick, MA). Volumes were obtained from the fast spin-echo multislice images based on regions of interest drawn about the tumor in each slice.

## 3. Results

### 3.1. Demonstration of Synergistic Combinations

**Figure 1:**
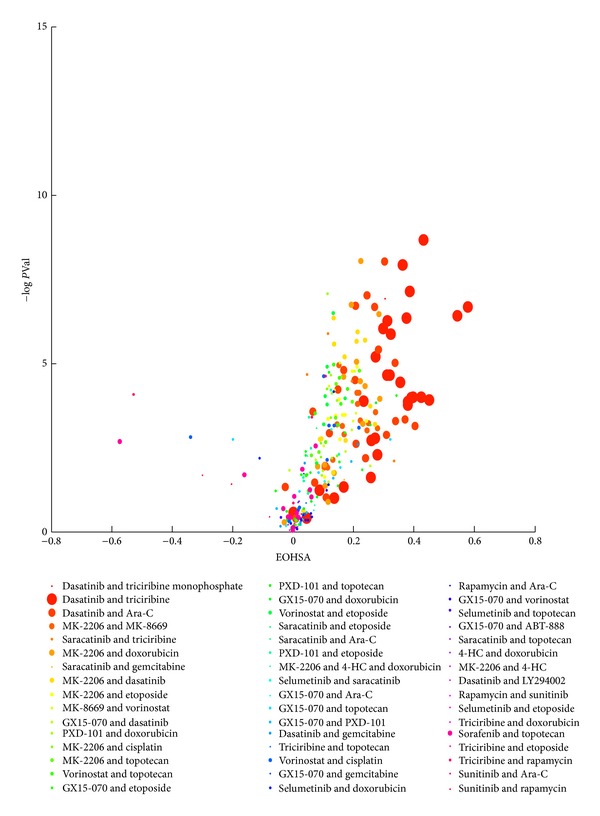
Mean excess over the highest single agent (EOHSA) versus *P* value for tested drug combinations in U2-OS osteosarcoma cells. EOHSA was calculated from cell viability assay dose response data for drug combinations and individual drugs for each combination. Results from combinations with potentially more significant synergy will show up in the upper right. Drug combinations are listed in the legend in order of the mean EOHSA value, with higher values appearing first. Symbol size represents the relative number of experiments included in the mean EOHSA value.

Many combinations of targeted agents and combinations of targeted and cytotoxic agents demonstrated synergy across multiple sarcoma cell lines. We used the EOHSA method to screen drug combination effects, as described in Materials and Methods. A representative volcano plot of synergy, expressed as EOHSA versus –log *P* value, is shown in Figure 1 (the average across all cell lines is shown in Supplementary any Material available online at http://dx.doi.org/10.1155/2013/365723 Figure S1). Combinations with promising synergy (i.e., higher EOHSA and –log *P* values) included mTOR inhibitors, HDAC inhibitors, and tyrosine kinase inhibitors, particularly those with Src and Akt activity. The complete combination effect data set for all 10 cell lines is shown in Supplementary any Material available online at http://dx.doi.org/10.1155/2013/365723 Tables S1 (concurrent treatment) and S2 (sequential treatment).

### 3.2. Histone Deacetylase Inhibitors and Topoisomerase II Inhibitors are Synergistic across a Variety of Sarcoma Cell Lines

Vorinostat (SAHA, suberoylanilide hydroxamic acid, Zolinza), a hydroxamate HDAC inhibitor, is particularly effective in inhibiting class I and II HDACs, more specifically HDAC1, HDAC2, HDAC3, and HDAC6 [6]. We found that vorinostat demonstrated single-agent activity in all 10 tested sarcoma cell lines. In pediatric-type cell lines, IC50 results ranged from 0.5 *μ*M in the RD-ES cell line to 4.3 *μ*M in the MNNG cell line, with a mean of 1.9 *μ*M (Table 2). In the adult-type sarcoma cell lines, IC50 results were lowest in the SW-872 cell line (2.5 *μ*M) and as high as 3.4 *μ*M in SK-UT-1 cells, with a mean of 2.9 *μ*M. These IC50 levels are within an order of magnitude of achievable levels in pediatric trials where the serum maximum concentration was 1 *μ*M [7].

**Figure 2:**
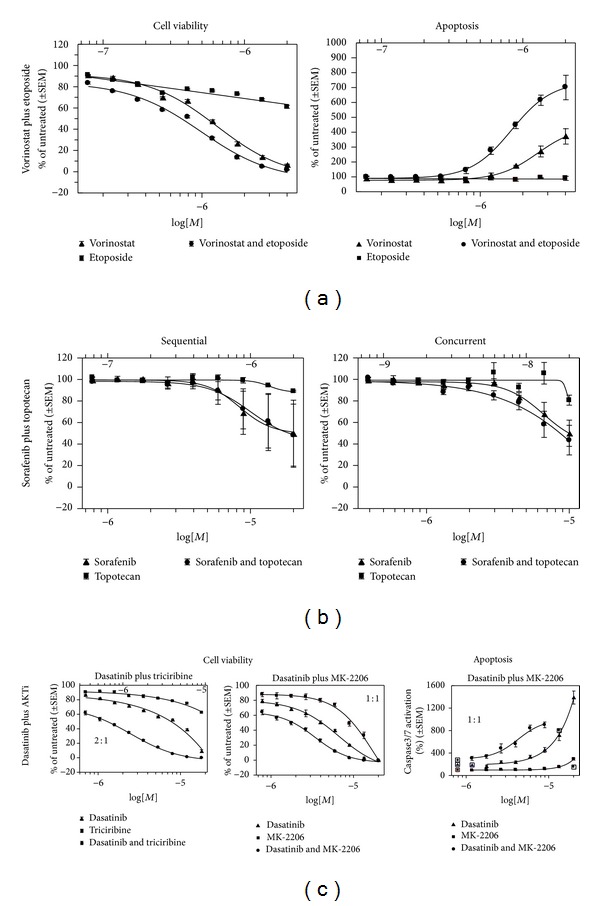
Drug combination effects in U2-OS osteosarcoma cells. The CellTiter-Blue assay was used to monitor viability in treated wells relative to untreated wells (% of untreated). Caspase 3/7 activation was measured after 24-hour concurrent treatments. (a) Vorinostat plus etoposide concurrent treatment. Cell viability was measured after 72-hour drug treatment. Vertical axis represents mean viability result for 5 independent experiments (*n* = 5). Apoptosis panel shows percent caspase 3/7 activation relative to untreated controls (100%). (b) Sorafenib plus topotecan concurrent and sequential drug treatment effects on cell viability. Sequential treatment consisted of a 24-hour pretreatment with sorafenib followed by the addition of topotecan. Cell viability was measured 48 hours after topotecan addition. (c) Dasatinib plus topotecan concurrent treatment. 72-hour cell viability assays were performed at constant drug ratios of 2 : 1 and 1 : 1. Caspase 3/7 activation was assayed after a 24-hour concurrent drug treatment (1 : 1 ratio) as in (a).

Topoisomerase II inhibitors such as etoposide also demonstrated broad activity. In the pediatric-type cell lines, IC50 results ranged from 0.5 *μ*M in the SK-ES-1 cell line to 6.8 *μ*M in the U2-OS cell line. In the adult-type sarcoma cell lines, IC50 results were lowest in the SK-UT-1 cell line at 2.5 *μ*M and as high as 7.4 *μ*M in HT-1080 cells (Table 2). Cells were also treated continuously with both agents for 72 hours at a constant 2 : 1 vorinostat: etoposide molar ratio. Using this combination, we found that 6 of 9 cell lines showed a synergistic interaction. The CI values for the pediatric cell lines ranged from 0.6 to 1.0 with a mean value of 0.8 (Table 2). The CI values for the adult-type cell lines ranged from 0.2 to 0.7 with a mean value of 0.5. The concurrent treatment of vorinostat and topotecan for 24 hours resulted in more than additive increases in caspase 3/7 activation, indicating that effects on viability are at least partially mediated through apoptosis (Figures 2(a) and 2(b)), as shown in the U2-OS cell line.

### 3.3. Tyrosine Kinases Have Varying Synergy with Topoisomerase I Inhibitors

Sorafenib is a multikinase inhibitor that affects specific targets involved in tumor cell proliferation [8]. Topotecan inhibits topoisomerase I and is currently being studied in a randomized controlled phase III study in Ewing sarcoma (ClinicalTrials.gov Identifier NCT01231906) with extensive use in a variety of pediatric tumors. 

We found that sorafenib demonstrated single-agent activity in all 5 tested sarcoma cells. In the pediatric-type cell lines, IC50 ranged from 2.6 *μ*M in the A-204 cell line to 8.0 *μ*M in the MNNG cell line, with a mean of 5.8 *μ*M (Table 2). Topotecan also demonstrated activity in all cell lines, with IC50 values ranging from 6.8 to 310 nM in pediatric sarcoma cell lines and from 73 to 400 nM in adult sarcoma cell lines (Table 2). Cells were also treated continuously with both sorafenib and topotecan for 72 hours in a 500 : 1 molar ratio, based on maximally achievable serum concentrations. This combination demonstrated a mix of additivity, synergy, and even some antagonism with the combination indices ranging from 0.53 in U2-OS cells to 1.7 in SK-ES-1 cells with an overall average of 1.05 (Table 2, Figures 2(c) and 2(d)).

### 3.4. Dasatinib and AKT Inhibitors Demonstrate Synergy across Many Sarcoma Cell Lines

Dasatinib is a targeted agent that inhibits multiple tyrosine kinases, including Src, Bcr-Abl, c-Kit, PDGFR*β*, and FGFR-1, at submicromolar concentrations [9–11]. Triciribine (API2) is a tricyclic nucleoside analogue that inhibits AKT1, -2, and -3 by interfering with membrane integration, which has been shown to inhibit AKT *in vivo* [12, 13]. MK-2206 is an allosteric inhibitor of the AKT kinase family of proteins (at nanomolar levels) without additional kinase inhibitory activity in a panel of 256 kinases [14].

Here, we found that dasatinib demonstrated single-agent activity in all 10 sarcoma cell lines. In the pediatric-type cell lines, IC50 results ranged from 4.2 *μ*M in the A-204 cell line to 12 *μ*M in the RD-ES cell line, with a mean of 7.6 *μ*M (Table 2). In the adult-type sarcomas, IC50 results were lowest in the SW-872 cell line at 4.6 *μ*M and as high as 9.9 *μ*M in SK-UT-1 cells, with a mean of 7.7 *μ*M. MK-2206 was also tested and demonstrated broad activity as well, with IC50 results ranging from 4.3 *μ*M in the SK-ES-1 cell line to 11 *μ*M in the U2-0S cell line, with a mean of 7.8 *μ*M (Table 2). In the adult-type sarcomas, IC50 results were lowest in the SK-LMS-1 cell line at 6.9 *μ*M and as high as 11 *μ*M in HT-1080 cells with a mean of 8.4 *μ*M. Triciribine demonstrated activity as well, with IC50 results ranging from 6.3 *μ*M in the RD-ES cell line to 69 *μ*M in the MNNG cell line with a mean of 24 *μ*M (Table 2). In the adult-type sarcomas, IC50 was lowest in the SK-LMS-1 cell line at 8.0 *μ*M and as high as 30 *μ*M in SK-UT-1 cells with a mean of 18 *μ*M.

Cells were also treated continuously with dasatinib and MK-2206 for 72 hours at a constant 1 : 1 molar ratio. All 10 cell lines demonstrated a synergistic interaction. The CI values for the pediatric cell lines ranged from 0.011 to 0.46 with a mean value of 0.19. The CI values for the adult-type cell lines ranged from 0.12 to 0.57 with a mean value of 0.39 (Table 2). 

**Figure 3:**
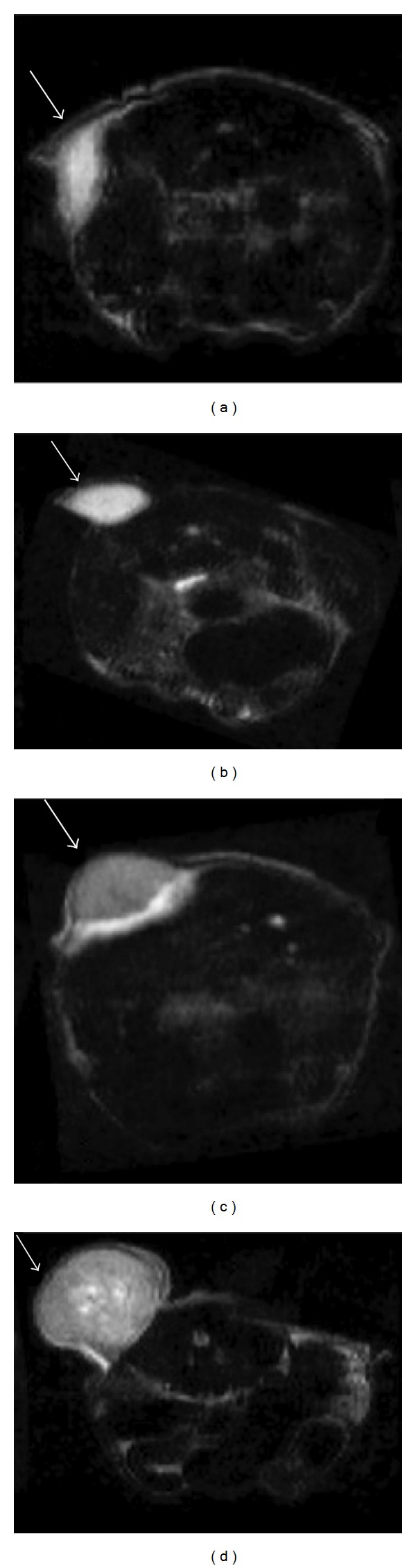
MRI imaging response to combination therapy in Ewing sarcoma. T2-weighted fast spin-echo images (TE/TR = 72/2400 ms) with a resolution of 312 mm demonstrating the tumor sizes in dasatinib (a), combination (b), triciribine (c), and untreated (d) at day 6. Representative datasets were chosen to reflect the overall trend of tumor size and growth. Lesions are indicated by arrows.

Cells were treated continuously with dasatinib and triciribine for 72 hours at a constant 2 : 1 molar ratio. All six tested cell lines indicated a synergistic interaction, with CI values for the pediatric cell lines ranging from 0.06 to 0.39 with a mean value of 0.24 (Table 2). The CI values for the two leiomyosarcoma cell lines were 0.24 and 0.27. The concurrent treatment of dasatinib with either MK-2206 or triciribine resulted in more than additive activity, and caspase 3/7 activation was also readily detected, indicating that effects on viability are at least partially mediated through apoptosis (Figure 3).

### 3.5. Dasatinib and Triciribine Have *In Vivo* Activity

Because of the robust synergy in the dasatinib and Akt inhibitor studies across sarcoma cell lines, we investigated the combination of dasatinib and triciribine *in vivo *using the A673 cell line. Tumors were evaluated for 3 weeks by caliper measurement and MRI.

**Figure 4:**
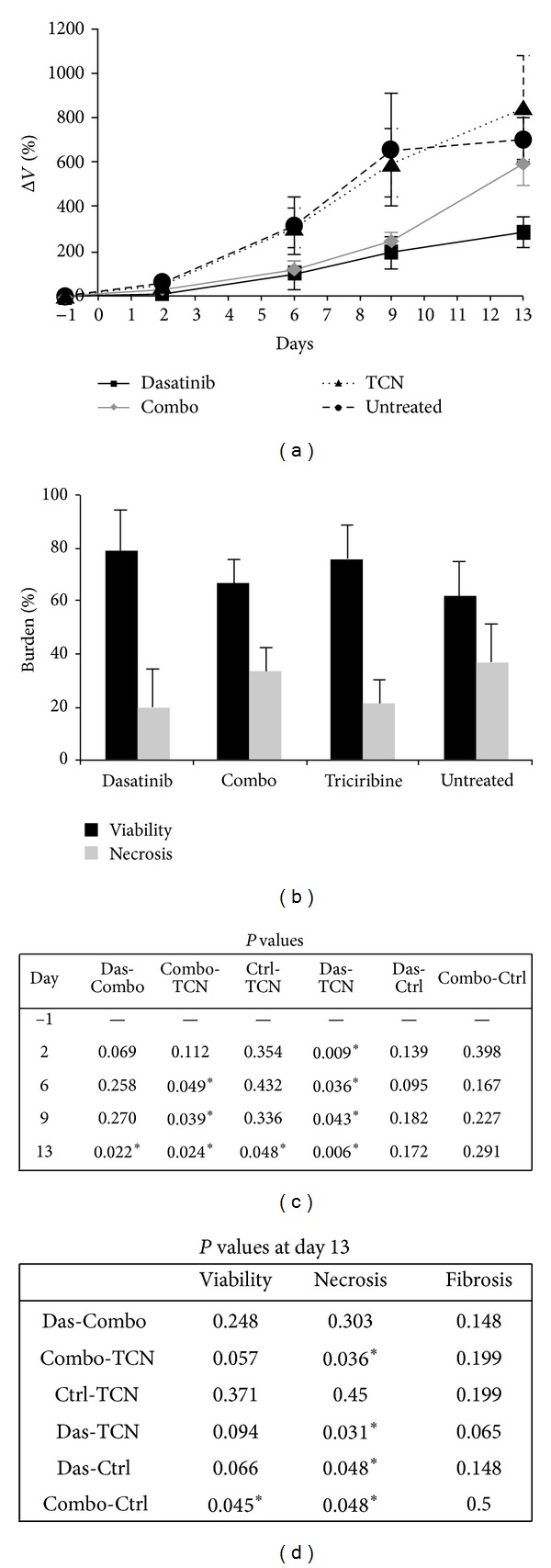
MRI tumor volume and pathologic response to combination therapy in Ewing sarcoma. (a) Region of interest analysis of T2-weighted MR datasets yielding the percent change in tumor size compared with that shown at initial value on day 0. (b) Quantitative histological results from H&E sections for viability, necrosis, and fibrosis at day 13. (c) *P* values comparing tumor volumes between treatment groups. (d) *P* values comparing pathologic determinants of response between treatment groups.

As measured by MRI, the relative change in tumor volume demonstrated similar tumor growth rates for all animals initially (Figure 4(b)). On day 6, the untreated control group and the triciribine-treated mice showed notable increases in tumor growth versus that shown in the dasatinib and combination groups (Figures 4(a)–4(c)). In fact, after the fourth treatment on day 2, dasatinib demonstrated significantly smaller tumor volumes than triciribine at all time points throughout the course of the experiment. Similarly, the combination group also showed significantly lower tumor volumes following the treatment on day 6 versus that shown in the triciribine and control groups. By day 13, the dasatinib group appeared to display the smallest tumor volumes, whereas triciribine did not appear to affect tumor growth. These results also agree with the MRI volume results (Figure 4(b)) and the histology H&E stains (Figure 4(c)).

Semiquantitative histological analyses of tumor tissue stained with H&E demonstrated an overall higher amount of necrosis in the combination and dasatinib groups than in any of the other groups (Figure 4(c)). Meanwhile, the untreated control and the triciribine only treated tumors displayed an overall lower amount of necrosis, which reached statistical significance compared with the dasatinib and the combination groups. Corroborating the volumetric data, we also observed significantly higher cell viability in the untreated versus the combination group.

## 4. Discussion

In this study, we systematically investigated a large number of agents and combinations from many classes in a relatively high-throughput fashion to determine synergistic combinations of chemotherapies for sarcomas. Due to the rarity of sarcomas, clinical trials are difficult to conduct, increasing the need for strong preclinical data to inform clinical trials. In addition, clinical attempts to modify chemotherapy and to develop new agents in sarcomas have been slow. 

The pediatric preclinical testing program (PPTP), a multi-institutional effort sponsored by the National Cancer Institute to evaluate new agents in pediatric malignancies using standardized *in vitro* and *in vivo* assays, has also tested agents across a variety of over 60 cell lines and xenografts [15, 16]. Through the PPTP, dasatinib, MK-2206, topotecan, sorafenib, and vorinostat have been tested [17–21], with results helping to inform single-agent, phase I pediatric studies. Our sarcoma cell line results were similar to the PPTP *in vitro* findings, with IC50 of >1 *μ*M for dasatinib, 0.5–10 *μ*M for vorinostat, around 10 *μ*M for MK-2206, and under the 10 *μ*M range for sorafenib [17, 18, 20, 21]. These micromolar IC50 levels are higher than the nanomolar IC50 for specific protein targets for tyrosine kinase inhibitors. Because of the manyfold higher concentrations leading to cell effects, the PPTP suggests off-target effects predominating [21]. The PPTP has demonstrated mostly low to intermediate *in vivo* activity of these compounds as single agents, although there was significant tumor growth delay in 4/5 tested osteosarcoma cells with sorafenib [18]. Topotecan had the most significant *in vivo* activity of the above compounds [19]. Importantly, although the PPTP has investigated combinations of drugs, the process is resource intensive [22].

The only FDA-approved agents for use in patients with front-line soft tissue sarcomas are doxorubicin and actinomycin D. Both have activity through topoisomerase II inhibition along with additional mechanisms of cellular toxicity. In this study, we tested additional topoisomerase I and II inhibitors, which may be given in select sarcomas and evaluated for synergy with a diverse collection of targeted agents. We also tested combinations of targeted agents and found multiple combinations that demonstrated synergy in our tested cell lines.

The antitumor effects of HDACs are largely thought to be related to effects on the 3-dimensional structure of DNA and effects on epigenetic modification of many genes; however, HDACs have other important roles in the cell, including roles in microtubule function, ubiquitination, and regulation of heat shock protein 90 [6]. Results from HDAC inhibitor and etoposide preclinical combination studies have contributed to a currently open pediatric phase I study (ClinicalTrials.gov Identifier NCI01294670) with vorinostat (SAHA) and etoposide. Since these data have been generated, there is increasing evidence of tolerability of HDACs in pediatric and adult patients. However, despite showing promise as a single agent, it has not led to phase II development, thus further validating the approach of looking for synergistic combinations [7, 23–25].

Other promising classes of compounds with demonstrated synergy are the tyrosine kinase inhibitors and topoisomerase inhibitors. As a class, tyrosine kinase inhibitors have an enormous variety in target specificity; thus it is not appropriate to consider them as one entity in terms of responses. In our study, dasatinib demonstrated synergy in most of our sarcoma models. Previous cell line experiments have shown that Src is activated in many sarcoma cells lines and that dasatinib can induce apoptosis and reduce cell migration [26, 27]. The dasatinib single-agent IC50 levels were more than an order of magnitude above achievable levels in pediatric trials, where the maximum concentration at the maximum tolerated dose was 0.3 *μ*M [28]. Among other targeted pathways screened, inhibitors of the PTEN/PI3K/AKT/mTOR pathway demonstrated a strong signal of synergy with dasatinib. This pathway plays an important role in tumor growth and survival and is mutated in many human cancers, including a subset of leiomyosarcomas and osteosarcomas [29–31]. Expression levels of phosphorylated Akt have been shown to have prognostic implications in a small series of extremity soft tissue sarcomas [32]. In our mouse xenograft study in the A673 Ewing sarcoma cell line, we found that the *in vivo* synergy was not dramatic in the dasatinib and triciribine combination group; however, this method could be used to assess other promising drug combinations.

Sorafenib's targets include the serine/threonine kinases c-Raf and B-Raf, the receptor tyrosine kinases RET, Flt-3, and c-Kit, and receptor tyrosine kinases important in tumor angiogenesis, including the vascular endothelial growth factor receptor family (VEGFR1, -2, and -3) and platelet-derived growth factor-beta [8]. The antitumor activity of sorafenib *in vivo* is driven by its direct effects on tumor growth through its inhibition of the Raf/MEK/ERK pathway and on the antiangiogenic activity of the compound. Because sorafenib has a stromal mechanism of action through VEGF inhibition, it was not expected that this assay would reflect all possible mechanisms of antitumor activity of this combination. Topotecan has established efficacy as a single agent or as part of a combination in a variety of pediatric malignancies, including germ cell tumors, Wilm's tumor, neuroblastoma, acute lymphoblastic leukemia, CNS malignancies, and sarcomas [33–41]. It has been given in multiple combinations with conventional chemotherapeutic agents, including alkylators, topoisomerase II inhibitors, topoisomerase I inhibitors, and microtubule inhibitors [34, 39, 42, 43].

We are currently exploring a combination of sorafenib and topotecan in pediatric solid tumor patients, recognizing that sorafenib functions also as an angiogenesis inhibitor (ClinicalTrials.gov Identifier NCT01683149). Promising activity, a progression-free survival of 20 weeks versus 7 weeks in placebo control, of another angiogenesis-inhibiting tyrosine kinase, pazopanib, has recently been demonstrated in selected subtypes of soft tissue sarcoma, leading to FDA approval of this agent in sarcomas, only the third agent with this disease indication [44]. Efficacy through angiogenesis or stromal mechanisms cannot be readily assessed in our *in vitro* assay. A recent study showed more striking activity of a pazopanib and topotecan *in vivo* than *in vitro *in several sarcoma models [45].

We also found that dasatinib combined with Akt inhibitors MK-2206 or triciribine demonstrated significant synergy across our cell lines, albeit at levels that were higher than those readily achievable in patient serum [13, 46]. We investigated the combination with *in vivo* testing through our xenograft model. Although tumor measurements did not decrease dramatically, we investigated imaging changes in this sarcoma model by MRI and observed pathologic changes in the tumor after therapy. Although this specific combination may be difficult to translate into clinical benefit, it does establish a translational approach that can be explored with other promising combinations.

While we present data on many combinations, there are inherent difficulties and limitations towards translation. As was recently published, sorafenib is heavily protein bound *in vivo* and vorinostat has a short life that does not lead to sustained 24-hour *in vivo* dose levels [47]. We also report variation in effects between sarcoma subtypes and within histologic subtypes. We think this likely represents the inherent tumor variation between patients and tumor heterogeneity within patient samples currently being described in sequencing efforts [48]. Our focus was to describe the effect of the combinations, and we did not explore thus far biomarkers of resistance or sensitivity. These insights are being more fully explored and will be a focus of this methodology as it goes forward.

## 5. Conclusion

The methods presented here demonstrate a comprehensive, reproducible, and high-throughput method for exploring antitumor effects of combinations of therapies. Combinations of targeted, targeted and cytotoxic, or multiple cytotoxic agents can be explored with this methodology. Combining more than two agents is also possible but requires different methodology when evaluating for synergy. This important early preclinical data can serve as the basis for confirmatory assays, xenograft studies, and ultimately clinical trials. Results from these efforts have contributed to preclinical data informing two active clinical trials in pediatrics.

## Supplementary Material

Supplemental Figure 1S: Mean excess over highest single agent (EOHSA) vs. *P* value for tested drug combinations in 10 sarcoma cell lines. EOHSA was calculated from cell viability assay dose-response data for drug combinations and individual drugs for each combination. Results from combinations with potentially more significant synergy will show up in the upper right quadrant. Drug combinations are listed in legend in order of the mean EOHSA value with higher values appearing first. Symbol size represents the relative number of experiments included in the mean EOHSA value.
Supplemental Table 1S: Concurrent 72 hour drug combination effect summary. The sarcoma cell line and molar drug ratio for each drug combination is indicated. Combination index (CI) values for effect levels of 0.75, 0.9 and 0.95 were calculated for each independent experiment by the method of Chou and Talalay and the mean CI value “CI(mean)” was calculated using the CI for each effect level. The standard error of the mean for CI values across independent experiments are shown “CI(SEM)”. EoHSA values and level of significance are represented in EoHSA (mean) and –log *P*-Value columns, respectively. Each line represents the calculated mean value for the number of independent experiments (*n*).
Supplemental Table 2S: Sequential drug combination effect summary. The sarcoma cell line and molar drug ratio for each drug combination is indicated. Time1 indicates first drug incubation time (hours) for first drug listed in combination. Time2 indicates incubation time (hours) for secondary drug(s) added. Combination index (CI) values for effect levels of 0.75, 0.9 and 0.95 were calculated for each independent experiment by the method of Chou and Talalay and the mean CI value “CI(mean)” was calculated using the CI for each effect level. The standard error of the mean for CI values across independent experiments are shown “CI(SEM)”. EoHSA values and level of significance are represented in EoHSA(mean) and –log *P*-Value columns, respectively. Each line represents the calculated mean value for the number of independent experiments (*n*).

## Figures and Tables

**Table 1:** Investigation, targeted agents used in study.

Agent	Mechanism of action	Source	Agreement
ABT-888	PARP inhibitor	AbbVie*	Single agent and with FDA approved
Dasatinib	Src, BCR-ABL inhibitor	BMS*	Single agent and with topoisomerase inhibitors or GX15-070
Etoposide	Topoisomerase II inhibitor	Commercial	FDA approved
GX15-070	BH3 mimetic	GeminX*	Single agent and with dasatinib or PXD101, and FDA approved
MK-2206	Akt inhibitor	Merck	Single agent and with dasatinib, MK-8669 or FDA approved
MK-8669	mTor inhibitor	Merck	Single agent and with dasatinib, MK-2206 or FDA approved
PXD101	HDAC inhibitor	CuraGen*	Single agent and with GX15-070
Saracatinib (AZD0530)	Src inhibitor	AstraZeneca*	Single agent and with saracatinib
Selumetinib (AZD6244, ARRY-142886)	MEK-1/2 inhibitor	AstraZeneca*	Single agent and with saracatinib and FDA approved
Sorafenib	VEGF, RAF inhibitor	Commercial	FDA approved
Topotecan	Topoisomerase I inhibitor	Commercial	FDA approved
Triciribine	AKT inhibitor, nucleoside	Commercial	FDA approved
Vorinostat	HDAC inhibitor	Merck*	FDA approved

*Obtained through CTEP N01-CM-62208.

**Table:** (a) Cell viability

Sarcoma type/cell line	Vorinostat			Etoposide			Sorafenib			Topotecan			Dasatinib			MK-2206			Triciribine		
	IC50 (*µ*M)	SEM	*n *	IC50 (*µ*M)	SEM	*n *	IC50 (*µ*M)	SEM	*n *	IC50 (nM)	SEM	*n *	IC50 (*µ*M)	SEM	*n *	IC50 (*µ*M)	SEM	*n *	IC50 (*µ*M)	SEM	*n *
Ewing																					
A-673	1.5	0.46	3	2.5	0.92	6				16	4.9	5	6.2	1.7	11				15	7.6	8
RD-ES	0.52		1	1.5	0.55	22	6.5	1.7	2	6.8	1.7	15	12	2.4	13	7.3	0.62	28	6.3	2.8	26
SK-ES-1	1.1	0.17	8	0.47	0.071	21	6.9	1.0	4	42	15	22	11	1.5	19	4.3	0.41	41	15	4.2	11
Osteosarcoma																					
MNNG HOS	4.3	1.2	4	5.6	2.0	15	8.0	0.71	3	310	140	20	5.8	0.77	22	9.2	0.55	40	69	27	3
U2-0S	1.6	0.25	8	6.8	1.1	14	4.9	0.75	2	190	56	7	6.4	0.76	19	11	0.72	29	19	5.0	11
Rhabdomyosarcoma																					
A-204	2.2	0.38	8	3.5	1.0	22	2.6	1.7	3	64	9.4	26	4.2	1.2	13	7.0	0.91	30	18	5.0	13
Fibrosarcoma																					
HT-1080	2.8	0.23	8	7.4	2.0	24				400	200	14	8.6	0.96	23	11	0.58	30	14	2.5	13
Leiomyosarcoma																					
SK-LMS-1	2.9	0.45	6	4.8	1.1	14				80	15	21	7.8	0.99	19	7.7	0.60	30	8.0	1.7	26
SK-UT-1	3.4	0.51	7	2.5	1.2	22				350	220	21	9.9	1.1	20	6.9	0.56	42	30	19	3
Liposarcoma																					
SW-872	2.5	0.41	7	2.9	0.84	21				73	27	21	4.6	0.72	23	7.9	0.57	41	18	3.6	7

**Table:** (b) Drug interaction measurements

Sarcoma type/cell line	Vorinostat and etoposide							Sorafenib and topotecan							Dasatinib and MK-2206							Dasatinib and triciribine						
	CI75	CI95	CI	SEM	*n*	EOHSA	neg log *P* Val	CI75	CI95	CI	SEM	*n*	EOHSA	neg log *P* Val	CI75	CI95	CI	SEM	*n*	EOHSA	neg log *P* Val	CI75	CI95	CI	SEM	*n*	EOHSA	neg log *P* Val
Ewing																												
A-673																						0.34	0.34	0.34	0.07	2	0.23	4.70
RD-ES								1.00	1.19	1.10	0.07	2	0.046	1.12	0.18	0.18	0.18	0.03	2	0.23	4.40	0.19	0.17	0.18	0.08	3	0.28	4.71
SK-ES-1	1.10	0.93	1.01	0.10	3	0.10	1.67	1.58	1.75	1.66	0.35	3	0.076	2.24	0.17	0.17	0.17	0.07	3	0.21	4.34	0.39	0.38	0.39	0.01	2	0.18	8.85
Osteosarcoma																												
MNNG HOS	0.78	0.95	0.86	0.27	3	0.08	1.62								0.022	0.003	0.011		1	0.17	4.10							
U2-OS	0.58	0.53	0.55	0.02	5	0.12	3.98	0.58	0.49	0.53	0.28	2	0.060	1.04	0.38	0.54	0.46	0.05	3	0.18	3.72	0.10	0.03	0.06		1	0.36	7.94
Rhabdoid																												
A-204	0.85	1.20	0.99	0.20	5	0.09	2.58	0.61	1.29	0.91	0.32	3	0.088	2.41	0.15	0.14	0.14		1	0.38	4.49							
Leiomyosarcoma																												
SK-LMS-1	0.69	0.68	0.67	0.10	3	0.09	2.23								0.31	0.34	0.32	0.03	3	0.21	3.69	0.24	0.23	0.24	0.05	3	0.27	4.61
SK-UT-1	0.73	0.68	0.70	0.17	3	0.11	5.39								0.12	0.12	0.12	0.10	2	0.27	7.09	0.28	0.25	0.27		1	0.18	3.82
Liposarcoma																												
SW-872	0.53	0.38	0.45	0.15	3	0.19	3.00								0.20	1.14	0.54	0.24	4	0.15	3.96							
Fibrosarcoma																												
HT-1080	0.23	0.16	0.19	0.04	5	0.24	2.79								0.39	0.76	0.57	0.17	3	0.18	2.87							
