# Supplementary material for: Rapid Screening of Novel Agents for Combination Therapy in Sarcomas
Source: Sarcoma. 2013 Oct 24;2013:365723. doi: 10.1155/2013/365723 (PMC3824404; doi:10.1155/2013/365723)

Supplemental Table 1S. Concurrent 72 hour drug combination effect summary. The sarcoma cell line and molar drug ratio for each drug combination is indicated. Combination index (CI) values for effect levels of 0.75, 0.9 and 0.95 were calculated for each independent experiment by the method of Chou and Talalay and the mean CI value "CI(mean)" was calculated using the CI for each effect level. The standard error of the mean for CI values across independent experiments are shown "CI(SEM)". EoHSA values and level of significance are represented in EoHSA(mean) and –log p-Value columns, respectively. Each line represents the calculated mean value for the number of independent experiments (n).

Sarcoma type	Cell line	Drug combination	Molar ratio	CI75	CI90	CI95	CI(mean)	CI(SEM)	n	EOHSA (mean)	neg log p-Value	n
Ewing	A-673	ABT-888 & Cisplatin	5	1.050	1.055	1.058	1.054		1	-0.031	1.04	1
Ewing	A-673	ABT-888 & Gemcitabine	250	1.050	1.028	1.013	1.030		1	0.111	2.41	1
Ewing	A-673	ABT-888 & Triciribine	2.5	1.246	1.240	1.235	1.241		1	0.051	2.45	1
Ewing	A-673	Dasatinib & Ara-C	1	0.205	0.203	0.202	0.203	0.040	2	0.170	5.06	2
Ewing	A-673	Dasatinib & Gemcitabine	10	0.637	0.615	0.601	0.618	0.062	2	0.157	2.77	2
Ewing	A-673	Dasatinib & Triciribine	0.1	0.418	0.415	0.413	0.416		1	0.258	4.86	1
Ewing	A-673	Dasatinib & Triciribine	0.2	0.619	0.613	0.609	0.614		1	0.150	4.67	1
Ewing	A-673	Dasatinib & Triciribine	1	0.176	0.165	0.157	0.166		1	0.230	4.05	1
Ewing	A-673	Dasatinib & Triciribine	2	0.338	0.339	0.340	0.339	0.066	2	0.229	4.70	2
Ewing	A-673	GX15-070 & Dasatinib	0.03	0.952	0.922	0.903	0.926	0.038	3	0.062	1.64	3
Ewing	A-673	GX15-070 & Doxorubicin	20	0.963	0.942	0.929	0.945		1	0.049	2.86	1
Ewing	A-673	GX15-070 & Etoposide	0.2	1.097	1.120	1.136	1.118		1	0.128	5.65	1
Ewing	A-673	GX15-070 & PXD-101	1	0.869	0.890	0.905	0.888	0.302	3	0.019	0.71	3
Ewing	A-673	GX15-070 & Vorinostat	0.03	1.142	1.140	1.139	1.140		1	-0.011	0.39	1
Ewing	A-673	GX15-070 & Vorinostat	0.06	1.251	1.240	1.233	1.241	0.123	2	0.032	1.58	2
Ewing	A-673	PXD-101 & Doxorubicin	2	0.901	0.897	0.894	0.897	0.069	2	0.037	0.34	1
Ewing	A-673	PXD-101 & Etoposide	0.1	0.821	0.813	0.807	0.814	0.133	2	0.099	1.42	1
Ewing	A-673	PXD-101 & Topotecan	1	1.044	1.048	1.050	1.048	0.028	2	0.047	2.65	1
Ewing	A-673	Rapamycin & Ara-C	10	0.776	0.877	0.953	0.868		1	0.063	2.01	1
Ewing	A-673	Rapamycin & Sunitinib	10	0.572	0.600	0.620	0.598		1	0.049	1.00	1
Ewing	A-673	Saracatinib & Ara-C	10	0.340	0.335	0.333	0.336		1	0.218	7.67	1
Ewing	A-673	Saracatinib & Gemcitabine	100	0.631	0.600	0.580	0.604		1	0.106	1.78	1
Ewing	A-673	Saracatinib & Triciribine	1	0.508	0.505	0.503	0.505		1	0.158	6.48	1
Ewing	A-673	Selumetinib & Doxorubicin	0.5	0.988	1.076	1.140	1.068		1	0.056	0.93	1
Ewing	A-673	Selumetinib & Doxorubicin	10	0.562	0.547	0.538	0.549		1	0.167	1.69	1
Ewing	A-673	Selumetinib & Etoposide	0.15	0.660	0.634	0.617	0.637		1	0.129	2.91	1
Ewing	A-673	Selumetinib & Etoposide	0.2	0.685	0.728	0.760	0.724		1	0.037	1.40	1
Ewing	A-673	Selumetinib & Topotecan	2	1.288	1.354	1.401	1.348		1	0.007	0.08	1
Ewing	A-673	Selumetinib & Topotecan	3	0.882	0.881	0.881	0.881		1	0.006	0.11	1
Ewing	A-673	Sunitinib & Ara-C	1	1.669	1.693	1.710	1.691		1	-0.017	0.63	1
Ewing	RD-ES	4-HC & Doxorubicin	50	1.752	1.733	1.720	1.735		1	-0.002	0.10	1
Ewing	RD-ES	Dasatinib & Ara-C	1	0.742	0.721	0.707	0.723	0.012	2	0.148	4.47	2
Ewing	RD-ES	Dasatinib & Gemcitabine	10	0.598	0.579	0.566	0.581		1	0.165	3.09	1
Ewing	RD-ES	Dasatinib & MK-2206	0.2	1.148	1.326	1.478	1.317	0.295	2	0.018	0.76	2
Ewing	RD-ES	Dasatinib & MK-2206	1	0.177	0.178	0.183	0.180	0.034	2	0.226	4.40	2
Ewing	RD-ES	Dasatinib & MK-2206	5	0.183	0.111	0.080	0.125		1	0.151	5.56	1
Ewing	RD-ES	Dasatinib & Triciribine	0.125	0.320	0.329	0.335	0.328		1	0.238	3.14	1
Ewing	RD-ES	Dasatinib & Triciribine	0.25	0.572	0.570	0.569	0.570	0.076	2	0.136	3.60	2
Ewing	RD-ES	Dasatinib & Triciribine	1	0.233	0.214	0.207	0.218	0.090	3	0.227	3.94	3
Ewing	RD-ES	Dasatinib & Triciribine	2	0.192	0.175	0.169	0.179	0.075	3	0.278	4.71	3
Ewing	RD-ES	GX15-070 & Dasatinib	0.03	0.681	0.618	0.578	0.626	0.127	2	0.091	2.49	2
Ewing	RD-ES	GX15-070 & Doxorubicin	2	1.117	1.106	1.099	1.107		1	0.019	1.78	1
Ewing	RD-ES	GX15-070 & Doxorubicin	20	1.267	1.269	1.270	1.268		1	0.073	1.31	1
Ewing	RD-ES	GX15-070 & Etoposide	0.2	0.656	0.711	0.752	0.706		1	0.096	1.26	1
Ewing	RD-ES	GX15-070 & PXD-101	1	1.207	1.137	1.093	1.146	0.028	3	-0.011	0.11	3
Ewing	RD-ES	MK-2206 & 4-HC	1	0.547	0.562	0.573	0.561		1	0.077	1.55	1
Ewing	RD-ES	MK-2206 & 4-HC & Doxorubicin	1	0.695	0.709	0.718	0.707		1	0.075	1.28	1
Ewing	RD-ES	MK-2206 & Cisplatin	0.5	0.948	1.143	1.349	1.122	0.254	4	0.081	1.36	4
Ewing	RD-ES	MK-2206 & Doxorubicin	100	0.534	0.471	0.439	0.481		1	0.204	3.20	1
Ewing	RD-ES	MK-2206 & Doxorubicin	20	0.443	0.408	0.392	0.414	0.084	5	0.195	4.47	5
Ewing	RD-ES	MK-2206 & Doxorubicin	50	0.502	0.489	0.480	0.490	0.111	2	0.166	2.18	2
Ewing	RD-ES	MK-2206 & Etoposide	1	0.213	0.239	0.260	0.237	0.079	5	0.144	3.42	5
Ewing	RD-ES	MK-2206 & Etoposide	5	0.449	0.448	0.475	0.457	0.087	5	0.130	1.49	5
Ewing	RD-ES	MK-2206 & MK-8669	0.2	0.589	0.549	0.542	0.560	0.126	9	0.208	4.04	8
Ewing	RD-ES	MK-2206 & MK-8669	2	0.072	0.177	0.370	0.206	0.102	3	0.257	3.89	2
Ewing	RD-ES	MK-2206 & Topotecan	50	0.606	0.616	0.627	0.616	0.021	4	0.045	0.85	4

Sarcoma type	Cell line	Drug combination	Molar ratio	CI75	CI90	CI95	CI(mean)	CI(SEM)	n	EOHSA (mean)	neg log p-Value	n
Ewing	RD-ES	MK-2206 & Topotecan	5000	0.383	0.308	0.359	0.350		1	0.017	0.39	1
Ewing	RD-ES	PXD-101 & Doxorubicin	2	0.980	0.994	1.004	0.992	0.063	2	0.092	1.87	2
Ewing	RD-ES	PXD-101 & Etoposide	0.1	1.073	1.080	1.085	1.079	0.237	2	0.056	1.82	2
Ewing	RD-ES	Saracatinib & Ara-C	10	1.030	1.007	0.993	1.010		1	0.111	2.94	1
Ewing	RD-ES	Saracatinib & Gemcitabine	100	1.320	1.283	1.259	1.287		1	-0.026	0.68	1
Ewing	RD-ES	Saracatinib & Triciribine	1	0.466	0.438	0.420	0.441		1	0.342	5.39	1
Ewing	RD-ES	Selumetinib & Doxorubicin	0.5	0.657	0.649	0.643	0.649		1	0.051	0.97	1
Ewing	RD-ES	Selumetinib & Doxorubicin	10	2.880	2.728	2.632	2.745		1	-0.071	0.76	1
Ewing	RD-ES	Selumetinib & Etoposide	0.2	0.486	0.471	0.462	0.473		1	0.093	1.93	1
Ewing	RD-ES	Selumetinib & Topotecan	2	1.399	1.384	1.374	1.386		1	-0.038	0.64	1
Ewing	RD-ES	Selumetinib & Topotecan	3	1.099	1.079	1.066	1.082		1	0.032	0.58	1
Ewing	RD-ES	Sorafenib & Topotecan	500	1.004	1.097	1.187	1.096	0.071	2	0.046	1.12	1
Ewing	RD-ES	Sunitinib & Ara-C	1	0.688	0.679	0.673	0.680		1	0.107	2.62	1
Ewing	RD-ES	Triciribine & Dasatinib	0.5	0.130	0.098	0.088	0.106	0.059	3	0.261	4.47	3
Ewing	RD-ES	Triciribine & Dasatinib	5	0.104	0.035	0.017	0.052		1	0.268	8.27	1
Ewing	RD-ES	Triciribine & Doxorubicin	800	1.288	1.285	1.284	1.285		1	0.034	1.28	1
Ewing	RD-ES	Triciribine & Etoposide	40	2.048	2.014	1.992	2.018		1	-0.054	1.38	1
Ewing	RD-ES	Triciribine & Rapamycin	0.2	0.746	0.550	0.452	0.583		1	0.051	1.50	1
Ewing	RD-ES	Triciribine & Rapamycin	1	2.618	31.708	173.240	24.319		1	0.063	3.80	1
Ewing	RD-ES	Triciribine & Rapamycin	5	0.900	0.826	0.782	0.836		1	0.005	0.36	1
Ewing	SK-ES-1	4-HC & Doxorubicin	50	1.103	1.089	1.079	1.090	0.027	2	0.015	0.81	2
Ewing	SK-ES-1	ABT-888 & Cisplatin	5	0.908	0.919	0.927	0.918		1	0.021	0.54	1
Ewing	SK-ES-1	ABT-888 & Gemcitabine	250	1.099	1.153	1.191	1.147		1	0.062	1.26	1
Ewing	SK-ES-1	ABT-888 & Triciribine	2.5	0.666	0.658	0.653	0.659		1	0.138	2.67	1
Ewing	SK-ES-1	Dasatinib & Ara-C	1	0.337	0.316	0.303	0.319		1	0.262	5.75	1
Ewing	SK-ES-1	Dasatinib & Ara-C	5	0.622	0.621	0.620	0.621		1	0.046	0.40	1
Ewing	SK-ES-1	Dasatinib & Doxorubicin	1	0.756	0.758	0.759	0.758		1	0.016	0.76	1
Ewing	SK-ES-1	Dasatinib & Doxorubicin	5	0.711	0.710	0.709	0.710		1	0.023	0.51	1
Ewing	SK-ES-1	Dasatinib & Etoposide	0.2	1.504	1.718	1.891	1.704		1			
Ewing	SK-ES-1	Dasatinib & Etoposide	1	0.998	1.197	1.366	1.178	0.127	5	0.035	0.56	2
Ewing	SK-ES-1	Dasatinib & Gemcitabine	10	0.626	0.638	0.646	0.637	0.077	3	0.080	1.10	3
Ewing	SK-ES-1	Dasatinib & MK-2206	0.1	0.539	0.541	0.551	0.543		1	0.124	2.08	1
Ewing	SK-ES-1	Dasatinib & MK-2206	0.2	0.887	0.827	0.826	0.846	0.327	3	0.075	1.46	3
Ewing	SK-ES-1	Dasatinib & MK-2206	1	0.167	0.167	0.168	0.167	0.070	3	0.215	4.34	3
Ewing	SK-ES-1	Dasatinib & MK-2206	5	0.308	0.381	0.459	0.383		1	0.205	6.90	1
Ewing	SK-ES-1	Dasatinib & Topotecan	10	0.635	0.667	0.690	0.664		1	0.047	0.94	1
Ewing	SK-ES-1	Dasatinib & Topotecan	100	1.004	1.362	1.684	1.350	0.034	2			
Ewing	SK-ES-1	Dasatinib & Topotecan	13.333	0.473	0.525	0.572	0.523		1			
Ewing	SK-ES-1	Dasatinib & Topotecan	500	1.201	1.676	2.141	1.627	0.072	2			
Ewing	SK-ES-1	Dasatinib & Triciribine	0.1	0.300	0.302	0.304	0.302	0.066	2	0.287	5.30	2
Ewing	SK-ES-1	Dasatinib & Triciribine	0.2	0.412	0.406	0.401	0.406		1	0.254	5.43	1
Ewing	SK-ES-1	Dasatinib & Triciribine	0.5	0.283	0.276	0.272	0.277	0.037	2	0.198	4.87	2
Ewing	SK-ES-1	Dasatinib & Triciribine	1	0.673	0.661	0.653	0.662	0.546	2	0.076	3.25	2
Ewing	SK-ES-1	Dasatinib & Triciribine	2	0.387	0.385	0.384	0.386	0.007	2	0.182	8.85	2
Ewing	SK-ES-1	GX15-070 & Dasatinib	0.03	0.935	0.908	0.891	0.911	0.012	2	0.073	1.48	2
Ewing	SK-ES-1	GX15-070 & PXD-101	1	1.305	1.402	1.476	1.395	0.311	2	0.028	0.65	2
Ewing	SK-ES-1	GX15-070 & Vorinostat	0.06	1.295	1.275	1.262	1.277	0.031	2	0.026	1.16	2
Ewing	SK-ES-1	MK-2206 & 4-HC	1	0.767	0.769	0.773	0.770	0.165	3	0.099	2.82	3
Ewing	SK-ES-1	MK-2206 & 4-HC & Doxorubicin	1	0.687	0.664	0.651	0.667	0.112	2	0.104	2.78	2
Ewing	SK-ES-1	MK-2206 & Cisplatin	0.5	1.006	1.028	1.056	1.030	0.088	3	0.075	2.50	3
Ewing	SK-ES-1	MK-2206 & Cisplatin	0.6667	1.206	1.244	1.285	1.245	0.024	4	0.066	3.58	4
Ewing	SK-ES-1	MK-2206 & Dasatinib	1	0.359	0.448	0.521	0.443		1	0.096	3.83	1
Ewing	SK-ES-1	MK-2206 & Doxorubicin	100	0.722	0.718	0.715	0.718		1	0.166	2.13	1
Ewing	SK-ES-1	MK-2206 & Doxorubicin	50	0.657	0.634	0.621	0.637	0.077	6	0.150	3.30	6
Ewing	SK-ES-1	MK-2206 & Etoposide	5	0.450	0.406	0.387	0.414	0.141	3	0.176	2.58	3
Ewing	SK-ES-1	MK-2206 & Etoposide	6.6667	0.258	0.197	0.173	0.209		1	0.306	4.57	1
Ewing	SK-ES-1	MK-2206 & MK-8669	0.2	0.755	0.931	1.293	0.943	0.131	10	0.160	4.50	7
Ewing	SK-ES-1	MK-2206 & MK-8669	2	0.855	0.924	0.997	0.925	0.774	2	0.184	7.22	2
Ewing	SK-ES-1	MK-2206 & Topotecan	50	0.514	0.544	0.580	0.546	0.058	3	0.120	2.17	3
Ewing	SK-ES-1	MK-2206 & Topotecan	500	0.309	0.275	0.280	0.288		1	0.134	3.48	1
Ewing	SK-ES-1	MK-2206 & Topotecan	66.667	0.831	1.210	1.653	1.181	0.297	3	0.078	1.58	3
Ewing	SK-ES-1	MK-8669 & MK-2206	0.2	0.814	0.765	0.734	0.771		1	0.123	1.39	1

Sarcoma type	Cell line	Drug combination	Molar ratio	CI75	CI90	CI95	CI(mean)	CI(SEM)	n	EOHSA (mean)	neg log p-Value	n
Ewing	SK-ES-1	MK-8669 & MK-2206	5	0.744	0.823	0.923	0.830	0.283	3			
Ewing	SK-ES-1	PXD-101 & Doxorubicin	2	0.925	0.924	0.924	0.924	0.152	2	0.166	3.67	1
Ewing	SK-ES-1	PXD-101 & Etoposide	0.1	0.879	0.879	0.879	0.879	0.047	2	0.108	3.61	1
Ewing	SK-ES-1	Saracatinib & Gemcitabine	100	0.914	0.937	0.953	0.935		1	0.084	1.19	1
Ewing	SK-ES-1	Saracatinib & Triciribine	1	0.370	0.352	0.340	0.354		1	0.233	4.15	1
Ewing	SK-ES-1	Selumetinib & Doxorubicin	0.5	0.933	0.979	1.012	0.975		1	0.047	0.94	1
Ewing	SK-ES-1	Selumetinib & Doxorubicin	10	0.837	0.745	0.690	0.758		1	0.162	1.26	1
Ewing	SK-ES-1	Selumetinib & Etoposide	0.15	0.991	1.027	1.052	1.024		1	0.010	0.46	1
Ewing	SK-ES-1	Selumetinib & Etoposide	0.2	1.071	1.100	1.130	1.100		1	0.011	0.47	1
Ewing	SK-ES-1	Selumetinib & Topotecan	2	0.504	0.550	0.586	0.547		1	0.019	0.47	1
Ewing	SK-ES-1	Selumetinib & Topotecan	3	0.877	0.808	0.764	0.816		1	0.014	0.21	1
Ewing	SK-ES-1	Sorafenib & Topotecan	50	2.522	3.165	3.731	3.087	2.180	4	-0.111	0.96	3
Ewing	SK-ES-1	Sorafenib & Topotecan	500	1.581	1.669	1.748	1.661	0.354	3	0.076	2.24	2
Ewing	SK-ES-1	Sunitinib & Ara-C	1	1.279	1.301	1.316	1.299		1	-0.045	3.06	1
Ewing	SK-ES-1	Vorinostat & Cisplatin	0.2	0.986	0.953	0.971	0.970	0.247	4	0.045	1.19	3
Ewing	SK-ES-1	Vorinostat & Etoposide	2	1.100	0.989	0.931	1.007	0.096	3	0.095	1.67	2
Ewing	SK-ES-1	Vorinostat & MK-8669	0.05	1.317	1.464	1.586	1.455		1			
Ewing	SK-ES-1	Vorinostat & Topotecan	8	0.783	0.729	0.789	0.767	0.127	2	0.017	1.42	1
osteosarcoma	MNNG HOS	4-HC & Doxorubicin	50	1.500	1.395	1.328	1.408		1	0.046	1.35	1
osteosarcoma	MNNG HOS	Dasatinib & Ara-C	5	0.184	0.169	0.160	0.171	0.007	2	0.287	4.66	2
osteosarcoma	MNNG HOS	Dasatinib & Doxorubicin	1	0.188	0.185	0.183	0.185		1	0.272	5.05	1
osteosarcoma	MNNG HOS	Dasatinib & Doxorubicin	5	0.196	0.225	0.247	0.223		1	0.162	4.90	1
osteosarcoma	MNNG HOS	Dasatinib & Etoposide	1	0.178	0.168	0.165	0.170	0.070	5	0.084	2.95	2
osteosarcoma	MNNG HOS	Dasatinib & Gemcitabine	10	0.063	0.058	0.054	0.058	0.020	2	0.251	5.35	2
osteosarcoma	MNNG HOS	Dasatinib & MK-2206	0.1	0.356	0.438	0.529	0.441		1	0.163	2.64	1
osteosarcoma	MNNG HOS	Dasatinib & MK-2206	0.2	1.153	1.737	2.395	1.686		1	0.068	1.24	1
osteosarcoma	MNNG HOS	Dasatinib & MK-2206	0.5	0.342	0.552	0.849	0.581		1	0.203	5.66	1
osteosarcoma	MNNG HOS	Dasatinib & MK-2206	1	0.022	0.006	0.003	0.011		1	0.173	4.10	1
osteosarcoma	MNNG HOS	Dasatinib & Topotecan	10	0.141	0.147	0.151	0.146	0.012	2	0.171	4.88	2
osteosarcoma	MNNG HOS	Dasatinib & Topotecan	100	0.208	0.210	0.223	0.214	0.169	3			
osteosarcoma	MNNG HOS	Dasatinib & Topotecan	20	0.934	0.996	1.041	0.990		1			
osteosarcoma	MNNG HOS	Dasatinib & Topotecan	500	0.499	0.245	0.151	0.298		1			
osteosarcoma	MNNG HOS	Dasatinib & Triciribine	0.1	0.715	0.699	0.689	0.701		1	0.023	0.60	1
osteosarcoma	MNNG HOS	Dasatinib & Triciribine	0.2	0.993	0.956	0.932	0.960		1	-0.019	0.37	1
osteosarcoma	MNNG HOS	GX15-070 & Dasatinib	0.03	0.873	0.873	0.872	0.873	0.099	2	0.114	2.98	2
osteosarcoma	MNNG HOS	GX15-070 & Doxorubicin	2	1.444	1.437	1.433	1.438		1	0.047	1.72	1
osteosarcoma	MNNG HOS	GX15-070 & PXD-101	1	1.470	1.512	1.541	1.507		1	-0.058	0.68	1
osteosarcoma	MNNG HOS	GX15-070 & Topotecan	1	1.333	1.347	1.356	1.345		1	0.072	2.67	1
osteosarcoma	MNNG HOS	GX15-070 & Vorinostat	0.03	1.540	1.521	1.508	1.523		1	-0.015	0.12	1
osteosarcoma	MNNG HOS	GX15-070 & Vorinostat	0.06	1.603	1.624	1.640	1.622	0.181	2	-0.075	0.77	2
osteosarcoma	MNNG HOS	MK-2206 & 4-HC	1	1.085	1.082	1.080	1.082		1	0.062	0.80	1
osteosarcoma	MNNG HOS	MK-2206 & 4-HC & Doxorubicin	1	0.690	0.681	0.676	0.682	0.223	2	0.115	2.54	2
osteosarcoma	MNNG HOS	MK-2206 & Cisplatin	0.5	2.603	3.931	5.289	3.779	0.440	2	0.026	1.22	2
osteosarcoma	MNNG HOS	MK-2206 & Dasatinib	1	0.210	0.229	0.273	0.237		1	0.204	4.02	1
osteosarcoma	MNNG HOS	MK-2206 & Doxorubicin	100	0.643	0.665	0.681	0.663		1	0.195	3.69	1
osteosarcoma	MNNG HOS	MK-2206 & Doxorubicin	5	0.665	0.653	0.646	0.655	0.058	3	0.166	4.29	3
osteosarcoma	MNNG HOS	MK-2206 & Doxorubicin	50	0.613	0.576	0.553	0.581	0.128	5	0.149	2.22	5
osteosarcoma	MNNG HOS	MK-2206 & Etoposide	5	0.490	0.432	0.401	0.441	0.097	3	0.165	1.94	3
osteosarcoma	MNNG HOS	MK-2206 & MK-8669	0.2	1.693	3.692	8.080	3.344	1.920	11	0.240	4.30	8
osteosarcoma	MNNG HOS	MK-2206 & Topotecan	50	1.603	3.279	5.686	3.068	2.892	2	0.036	0.91	2
osteosarcoma	MNNG HOS	MK-8669 & MK-2206	5	1.177	1.259	1.331	1.256	0.083	3			
osteosarcoma	MNNG HOS	PXD-101 & Doxorubicin	2	1.508	1.488	1.476	1.491		1			
osteosarcoma	MNNG HOS	PXD-101 & Etoposide	0.1	1.034	1.039	1.043	1.039		1			
osteosarcoma	MNNG HOS	PXD-101 & Topotecan	1	0.953	0.934	0.922	0.936		1			
osteosarcoma	MNNG HOS	Saracatinib & Ara-C	10	0.557	0.564	0.569	0.563		1	0.153	3.41	1
osteosarcoma	MNNG HOS	Saracatinib & Gemcitabine	100	0.572	0.558	0.549	0.560		1	0.203	2.98	1
osteosarcoma	MNNG HOS	Selumetinib & Doxorubicin	0.5	0.912	0.909	0.906	0.909		1	-0.096	0.52	1
osteosarcoma	MNNG HOS	Selumetinib & Doxorubicin	10	0.425	0.350	0.307	0.361		1	0.141	1.01	1
osteosarcoma	MNNG HOS	Selumetinib & Etoposide	0.15	0.981	0.997	1.007	0.995		1	0.057	2.20	1
osteosarcoma	MNNG HOS	Selumetinib & Etoposide	0.2	0.828	0.886	0.927	0.880		1	0.005	0.08	1
osteosarcoma	MNNG HOS	Selumetinib & Topotecan	2	1.053	1.060	1.065	1.059		1	0.020	0.72	1

Sarcoma type	Cell line	Drug combination	Molar ratio	CI75	CI90	CI95	CI(mean)	CI(SEM)	n	EOHSA (mean)	neg log p-Value	n
osteosarcoma	MNNG HOS	Selumetinib & Topotecan	3	0.758	0.746	0.738	0.747		1	0.030	1.08	1
osteosarcoma	MNNG HOS	Sorafenib & Topotecan	50	1.012	1.002	1.000	1.005		1			
osteosarcoma	MNNG HOS	Sunitinib & Ara-C	1	0.971	0.971	0.971	0.971		1	-0.028	0.32	1
osteosarcoma	MNNG HOS	Sunitinib & Ara-C	2	0.861	0.870	0.876	0.869		1	0.090	3.01	1
osteosarcoma	MNNG HOS	Vorinostat & Cisplatin	0.2	0.981	0.946	0.927	0.951	0.165	3	0.042	1.17	3
osteosarcoma	MNNG HOS	Vorinostat & Etoposide	2	0.779	0.861	0.951	0.864	0.274	3	0.076	1.62	2
osteosarcoma	MNNG HOS	Vorinostat & MK-8669	0.05	0.990	0.992	0.995	0.992	0.496	1			
osteosarcoma	MNNG HOS	Vorinostat & Topotecan	8	0.946	0.938	0.940	0.941	0.118	4	0.061	1.82	3
osteosarcoma	U2-OS	Dasatinib & Ara-C	1	0.497	0.485	0.478	0.487		1	0.105	1.97	1
osteosarcoma	U2-OS	Dasatinib & Gemcitabine	10	0.368	0.367	0.367	0.367		1	0.213	2.61	1
osteosarcoma	U2-OS	Dasatinib & MK-2206	0.2	1.304	2.091	3.050	2.005	0.655	3	0.126	2.78	3
osteosarcoma	U2-OS	Dasatinib & MK-2206	1	0.379	0.463	0.542	0.461	0.045	3	0.176	3.72	3
osteosarcoma	U2-OS	Dasatinib & Triciribine	0.1	0.353	0.367	0.377	0.366		1	0.270	2.78	1
osteosarcoma	U2-OS	Dasatinib & Triciribine	0.25	0.078	0.076	0.074	0.076		1	0.424	4.00	1
osteosarcoma	U2-OS	Dasatinib & Triciribine	0.5	0.068	0.064	0.061	0.064		1	0.451	3.92	1
osteosarcoma	U2-OS	Dasatinib & Triciribine	1	0.098	0.078	0.070	0.082	0.009	2	0.350	5.47	2
osteosarcoma	U2-OS	Dasatinib & Triciribine	2	0.097	0.049	0.031	0.059		1	0.361	7.94	1
osteosarcoma	U2-OS	GX15-070 & Dasatinib	0.03	0.786	0.775	0.768	0.776		1	0.116	1.50	1
osteosarcoma	U2-OS	GX15-070 & Doxorubicin	20	0.490	0.473	0.462	0.475	0.165	2	0.068	1.12	2
osteosarcoma	U2-OS	GX15-070 & Etoposide	0.2	0.810	0.763	0.734	0.769	0.304	3	0.139	1.68	3
osteosarcoma	U2-OS	GX15-070 & PXD-101	1	1.042	1.020	1.005	1.022		1	0.133	1.60	1
osteosarcoma	U2-OS	MK-2206 & Cisplatin	0.5	1.521	1.793	2.007	1.762		1	0.092	2.65	1
osteosarcoma	U2-OS	MK-2206 & Doxorubicin	100	0.561	0.543	0.532	0.545		1	0.192	6.75	1
osteosarcoma	U2-OS	MK-2206 & Doxorubicin	20	0.452	0.435	0.433	0.440	0.095	5	0.228	4.12	5
osteosarcoma	U2-OS	MK-2206 & Doxorubicin	5	0.514	0.511	0.510	0.512	0.062	2	0.119	1.87	2
osteosarcoma	U2-OS	MK-2206 & Doxorubicin	50	0.423	0.414	0.408	0.415	0.107	3	0.239	6.52	3
osteosarcoma	U2-OS	MK-2206 & Etoposide	1	0.606	0.659	0.710	0.658	0.137	4	0.187	3.88	4
osteosarcoma	U2-OS	MK-2206 & Etoposide	5	0.209	0.197	0.188	0.198		1	0.211	4.92	1
osteosarcoma	U2-OS	MK-2206 & MK-8669	0.2	0.749	7.622	132.156	5.276	4.535	9	0.205	3.21	9
osteosarcoma	U2-OS	MK-2206 & MK-8669	2	0.574	32.659	510.464	21.226		1	0.211	4.13	1
osteosarcoma	U2-OS	PXD-101 & Doxorubicin	2	0.919	0.976	1.017	0.970		1	0.094	1.69	1
osteosarcoma	U2-OS	PXD-101 & Etoposide	0.1	0.463	0.450	0.442	0.452		1	0.151	2.31	1
osteosarcoma	U2-OS	Rapamycin & Sunitinib	10	1.114	1.114	1.115	1.114		1	0.000	0.14	1
osteosarcoma	U2-OS	Selumetinib & Doxorubicin	0.5	0.761	0.793	0.815	0.790		1	0.061	0.45	1
osteosarcoma	U2-OS	Selumetinib & Doxorubicin	10	1.013	1.079	1.128	1.073		1	0.116	1.40	1
osteosarcoma	U2-OS	Selumetinib & Etoposide	0.2	1.368	1.355	1.347	1.357		1	0.014	0.15	1
osteosarcoma	U2-OS	Selumetinib & Topotecan	2	0.787	0.802	0.814	0.801		1	0.110	1.23	1
osteosarcoma	U2-OS	Sorafenib & Topotecan	50	0.813	0.810	0.870	0.831	0.359	3	0.022	0.84	2
osteosarcoma	U2-OS	Sorafenib & Topotecan	500	0.583	0.514	0.485	0.527	0.277	2	0.060	1.04	1
osteosarcoma	U2-OS	Sunitinib & Rapamycin	3.75	3.590	3.418	3.307	3.436		1	-0.205	1.43	1
osteosarcoma	U2-OS	Triciribine & Dasatinib	0.5	0.097	0.087	0.081	0.088	0.029	2	0.476	6.52	2
osteosarcoma	U2-OS	Triciribine & Topotecan	400	1.718	1.841	1.931	1.830		1	0.043	0.87	1
osteosarcoma	U2-OS	Vorinostat & Cisplatin	0.2	2.264	2.874	3.467	2.805	2.309	5	0.048	2.88	5
osteosarcoma	U2-OS	Vorinostat & Etoposide	2	0.582	0.548	0.530	0.553	0.019	5	0.120	3.98	5
osteosarcoma	U2-OS	Vorinostat & MK-8669	0.05	1.052	1.218	1.366	1.198	0.487	2	0.150	2.93	2
osteosarcoma	U2-OS	Vorinostat & Topotecan	8	0.542	0.514	0.498	0.518	0.049	5	0.137	4.10	5
rhabdomyosarcoma	A-204	Dasatinib & Gemcitabine	25	0.665	0.620	0.592	0.626		1	0.243	4.68	1
rhabdomyosarcoma	A-204	Dasatinib & Gemcitabine	50	0.542	0.489	0.456	0.496		1	0.116	3.38	1
rhabdomyosarcoma	A-204	Dasatinib & MK-2206	1	0.150	0.140	0.137	0.142		1	0.381	4.49	1
rhabdomyosarcoma	A-204	Dasatinib & Triciribine	0.1	0.650	0.672	0.687	0.669	0.078	2	0.143	2.76	2
rhabdomyosarcoma	A-204	Dasatinib & Triciribine	0.125	0.325	0.310	0.300	0.312		1	0.183	3.85	1
rhabdomyosarcoma	A-204	GX15-070 & Dasatinib	0.03	0.393	0.356	0.334	0.361		1	0.163	2.82	1
rhabdomyosarcoma	A-204	GX15-070 & Doxorubicin	20	0.620	0.603	0.592	0.605	0.141	3	0.173	3.68	3
rhabdomyosarcoma	A-204	GX15-070 & Etoposide	0.2	1.225	1.177	1.148	1.183	0.094	2	0.067	1.04	2
rhabdomyosarcoma	A-204	GX15-070 & Topotecan	10	0.833	0.836	0.838	0.836	0.556	2	0.088	1.49	2
rhabdomyosarcoma	A-204	MK-2206 & Cisplatin	0.5	1.057	1.148	1.337	1.181		1	0.076	2.37	1
rhabdomyosarcoma	A-204	MK-2206 & Doxorubicin	20	0.559	0.593	0.628	0.593	0.097	5	0.249	3.17	5
rhabdomyosarcoma	A-204	MK-2206 & Etoposide	1	0.523	0.552	0.579	0.551	0.106	4	0.204	2.97	4
rhabdomyosarcoma	A-204	MK-2206 & Etoposide	5	0.952	0.959	0.970	0.960		1	0.081	2.33	1
rhabdomyosarcoma	A-204	MK-2206 & MK-8669	0.2	0.663	0.695	0.740	0.700	0.074	7	0.212	3.63	7
rhabdomyosarcoma	A-204	MK-2206 & MK-8669	2	0.342	0.572	0.810	0.575		1	0.154	3.39	1

Sarcoma type	Cell line	Drug combination	Molar ratio	CI75	CI90	CI95	CI(mean)	CI(SEM)	n	EOHSA (mean)	neg log p-Value	n
rhabdomyosarcoma	A-204	MK-2206 & Topotecan	50	1.163	2.333	4.029	2.219		1	0.113	2.44	1
rhabdomyosarcoma	A-204	PXD-101 & Doxorubicin	2	0.902	0.893	0.887	0.894		1	0.075	2.34	1
rhabdomyosarcoma	A-204	PXD-101 & Topotecan	1	1.318	1.281	1.257	1.286		1	-0.016	0.21	1
rhabdomyosarcoma	A-204	Selumetinib & Doxorubicin	0.5	0.695	0.720	0.738	0.718		1	0.061	1.05	1
rhabdomyosarcoma	A-204	Selumetinib & Doxorubicin	10	1.385	1.439	1.476	1.433		1	0.017	0.35	1
rhabdomyosarcoma	A-204	Selumetinib & Etoposide	0.15	1.465	1.351	1.278	1.364		1	0.020	0.69	1
rhabdomyosarcoma	A-204	Selumetinib & Topotecan	2	1.996	2.117	2.204	2.104		1	0.004	0.18	1
rhabdomyosarcoma	A-204	Selumetinib & Topotecan	3	1.070	1.055	1.046	1.057		1	0.030	1.37	1
rhabdomyosarcoma	A-204	Sorafenib & Topotecan	50	0.527	0.763	1.088	0.730	0.381	3	0.149	4.41	2
rhabdomyosarcoma	A-204	Sorafenib & Topotecan	500	0.611	0.922	1.291	0.913	0.318	3	0.088	2.41	2
rhabdomyosarcoma	A-204	Triciribine & Dasatinib	0.5	1.152	0.184	0.053	0.463		1	-0.031	0.47	1
rhabdomyosarcoma	A-204	Triciribine & Rapamycin	0.2	0.275	0.181	0.137	0.198		1	0.084	2.64	1
rhabdomyosarcoma	A-204	Triciribine & Topotecan	400	0.374	0.349	0.334	0.352		1	0.115	3.57	1
rhabdomyosarcoma	A-204	Vorinostat & Cisplatin	0.2	1.652	2.105	2.546	2.072	1.147	5	0.098	3.02	5
rhabdomyosarcoma	A-204	Vorinostat & Etoposide	2	0.847	0.973	1.204	0.993	0.203	5	0.094	2.58	5
rhabdomyosarcoma	A-204	Vorinostat & MK-8669	0.05	0.911	0.864	0.837	0.870	0.025	2	0.078	1.31	1
rhabdomyosarcoma	A-204	Vorinostat & MK-8669	0.5	3.730	8.169	13.939	7.517		1			
rhabdomyosarcoma	A-204	Vorinostat & Topotecan	8	0.920	1.703	3.158	1.640	0.278	5	0.075	2.24	5
leiomyosarcoma	SK-LMS-1	4-HC & Doxorubicin	50	1.223	1.181	1.155	1.186		1	-0.018	0.13	1
leiomyosarcoma	SK-LMS-1	Dasatinib & Ara-C	0.2	0.194	0.205	0.213	0.204	0.044	2	0.160	3.69	2
leiomyosarcoma	SK-LMS-1	Dasatinib & Ara-C	1	0.483	0.489	0.495	0.489	0.138	3	0.160	2.97	3
leiomyosarcoma	SK-LMS-1	Dasatinib & Ara-C	5	0.308	0.291	0.281	0.293		1	0.264	2.61	1
leiomyosarcoma	SK-LMS-1	Dasatinib & Gemcitabine	50	1.068	1.096	1.116	1.093		1	0.087	1.06	1
leiomyosarcoma	SK-LMS-1	Dasatinib & MK-2206	0.2	0.922	0.877	0.858	0.886	0.243	3	0.038	1.22	3
leiomyosarcoma	SK-LMS-1	Dasatinib & MK-2206	1	0.314	0.321	0.336	0.324	0.034	3	0.208	3.69	3
leiomyosarcoma	SK-LMS-1	Dasatinib & Triciribine	0.1	0.380	0.393	0.402	0.391	0.094	2	0.211	2.49	2
leiomyosarcoma	SK-LMS-1	Dasatinib & Triciribine	0.125	0.370	0.351	0.339	0.353		1	0.158	3.06	1
leiomyosarcoma	SK-LMS-1	Dasatinib & Triciribine	0.25	0.318	0.314	0.312	0.315		1	0.292	5.57	1
leiomyosarcoma	SK-LMS-1	Dasatinib & Triciribine	0.5	0.765	0.769	0.771	0.768		1	0.231	3.37	1
leiomyosarcoma	SK-LMS-1	Dasatinib & Triciribine	1	0.250	0.243	0.238	0.244	0.089	2	0.279	3.67	2
leiomyosarcoma	SK-LMS-1	Dasatinib & Triciribine	2	0.241	0.235	0.232	0.236	0.045	3	0.273	4.61	3
leiomyosarcoma	SK-LMS-1	GX15-070 & Dasatinib	0.03	1.077	1.071	1.067	1.072	0.126	3	0.061	1.33	3
leiomyosarcoma	SK-LMS-1	GX15-070 & Doxorubicin	20	0.894	0.872	0.857	0.875	0.084	2	0.094	2.18	2
leiomyosarcoma	SK-LMS-1	GX15-070 & Etoposide	0.2	0.603	0.612	0.619	0.611	0.106	2	0.166	2.38	2
leiomyosarcoma	SK-LMS-1	GX15-070 & PXD-101	1	1.094	1.077	1.066	1.079		1	0.013	0.24	1
leiomyosarcoma	SK-LMS-1	GX15-070 & Topotecan	10	1.310	1.275	1.253	1.279	0.566	3	0.079	4.05	3
leiomyosarcoma	SK-LMS-1	MK-2206 & 4-HC	1	0.796	0.787	0.781	0.788		1	0.025	0.65	1
leiomyosarcoma	SK-LMS-1	MK-2206 & 4-HC & Doxorubicin	1	0.821	0.777	0.749	0.782		1	0.078	2.55	1
leiomyosarcoma	SK-LMS-1	MK-2206 & Cisplatin	0.5	0.944	1.384	1.850	1.333	0.548	2	0.142	4.61	2
leiomyosarcoma	SK-LMS-1	MK-2206 & Doxorubicin	100	0.870	0.710	0.636	0.739		1	0.088	2.54	1
leiomyosarcoma	SK-LMS-1	MK-2206 & Doxorubicin	20	0.693	0.694	0.704	0.697	0.088	5	0.154	2.92	5
leiomyosarcoma	SK-LMS-1	MK-2206 & Doxorubicin	5	0.386	0.397	0.404	0.396		1	0.185	3.75	1
leiomyosarcoma	SK-LMS-1	MK-2206 & Doxorubicin	50	0.735	0.706	0.688	0.709	0.089	4	0.094	2.33	4
leiomyosarcoma	SK-LMS-1	MK-2206 & Etoposide	1	0.611	0.595	0.589	0.598	0.074	5	0.158	3.00	5
leiomyosarcoma	SK-LMS-1	MK-2206 & Etoposide	5	0.350	0.308	0.286	0.315	0.234	3	0.186	3.88	3
leiomyosarcoma	SK-LMS-1	MK-2206 & MK-8669	0.2	0.813	0.819	0.897	0.843	0.126	10	0.159	3.37	9
leiomyosarcoma	SK-LMS-1	MK-2206 & MK-8669	2	0.906	0.928	0.946	0.927		1			
leiomyosarcoma	SK-LMS-1	MK-2206 & Topotecan	50	0.306	0.293	0.297	0.299	0.029	2	0.118	3.67	2
leiomyosarcoma	SK-LMS-1	Saracatinib & Gemcitabine	100	1.884	1.897	1.906	1.896		1	-0.002	0.04	1
leiomyosarcoma	SK-LMS-1	Saracatinib & Triciribine	1	0.757	0.759	0.760	0.759		1	0.198	2.63	1
leiomyosarcoma	SK-LMS-1	Selumetinib & Doxorubicin	10	1.237	1.234	1.232	1.235		1	0.077	0.98	1
leiomyosarcoma	SK-LMS-1	Selumetinib & Topotecan	2	0.998	0.998	0.999	0.998		1	0.082	1.51	1
leiomyosarcoma	SK-LMS-1	Triciribine & Dasatinib	0.5	0.090	0.061	0.064	0.072	0.025	3	0.286	5.89	3
leiomyosarcoma	SK-LMS-1	Triciribine & Dasatinib	5	0.024	0.004	0.001	0.009		1	0.163	1.45	1
leiomyosarcoma	SK-LMS-1	Triciribine & Doxorubicin	800	1.220	1.218	1.216	1.218		1	-0.012	0.08	1
leiomyosarcoma	SK-LMS-1	Triciribine & Etoposide	40	2.977	2.949	2.930	2.952		1	-0.120	1.94	1
leiomyosarcoma	SK-LMS-1	Triciribine & Rapamycin	5	2.030	3.044	4.036	2.922		1	0.014	0.12	1
leiomyosarcoma	SK-LMS-1	Triciribine & Topotecan	400	0.858	0.835	0.820	0.838		1	0.044	3.78	1
leiomyosarcoma	SK-LMS-1	Vorinostat & Cisplatin	0.2	0.391	0.285	0.238	0.304	0.073	4	0.132	1.88	4
leiomyosarcoma	SK-LMS-1	Vorinostat & Etoposide	2	0.686	0.656	0.675	0.672	0.100	3	0.093	2.23	3
leiomyosarcoma	SK-LMS-1	Vorinostat & MK-8669	0.05	1.323	1.616	1.945	1.587	0.806	2	0.011	0.24	2

Sarcoma type	Cell line	Drug combination	Molar ratio	CI75	CI90	CI95	CI(mean)	CI(SEM)	n	EOHSA (mean)	neg log p-Value	n
leiomyosarcoma	SK-LMS-1	Vorinostat & Topotecan	8	1.043	1.556	2.482	1.521	0.673	4	0.021	0.60	4
leiomyosarcoma	SK-UT-1	4-HC & Doxorubicin	50	1.215	1.186	1.168	1.190	0.215	2	0.022	0.74	2
leiomyosarcoma	SK-UT-1	ABT-888 & Cisplatin	5	1.192	1.238	1.270	1.234		1	0.051	1.89	1
leiomyosarcoma	SK-UT-1	ABT-888 & Gemcitabine	250	0.985	0.987	0.989	0.987		1	0.039	1.78	1
leiomyosarcoma	SK-UT-1	Dasatinib & Ara-C	1	0.157	0.149	0.143	0.150		1	0.214	8.70	1
leiomyosarcoma	SK-UT-1	Dasatinib & Ara-C	5	0.230	0.228	0.227	0.228		1	0.314	5.04	1
leiomyosarcoma	SK-UT-1	Dasatinib & Doxorubicin	1	0.415	0.386	0.367	0.389		1	0.120	4.48	1
leiomyosarcoma	SK-UT-1	Dasatinib & Doxorubicin	5	0.400	0.399	0.397	0.399		1	0.137	3.11	1
leiomyosarcoma	SK-UT-1	Dasatinib & Etoposide	0.2	0.091	0.210	0.380	0.227		1			
leiomyosarcoma	SK-UT-1	Dasatinib & Etoposide	1	0.412	0.412	0.412	0.412	0.047	3	0.105	1.58	2
leiomyosarcoma	SK-UT-1	Dasatinib & Gemcitabine	10	0.269	0.262	0.258	0.263		1	0.134	3.00	1
leiomyosarcoma	SK-UT-1	Dasatinib & MK-2206	0.1	0.298	0.270	0.256	0.275		1	0.142	2.67	1
leiomyosarcoma	SK-UT-1	Dasatinib & MK-2206	0.2	0.673	0.601	0.573	0.616	0.311	2	0.104	2.29	2
leiomyosarcoma	SK-UT-1	Dasatinib & MK-2206	0.5	0.237	0.226	0.223	0.229		1	0.216	4.58	1
leiomyosarcoma	SK-UT-1	Dasatinib & MK-2206	1	0.120	0.119	0.121	0.120	0.095	2	0.265	7.09	2
leiomyosarcoma	SK-UT-1	Dasatinib & MK-2206	2.5	0.076	0.049	0.037	0.054		1	0.234	10.60	1
leiomyosarcoma	SK-UT-1	Dasatinib & MK-2206	5	0.106	0.101	0.108	0.105	0.076	2	0.246	7.81	2
leiomyosarcoma	SK-UT-1	Dasatinib & Topotecan	1.3333	0.003	0.001	0.000	0.001		1			
leiomyosarcoma	SK-UT-1	Dasatinib & Topotecan	10	0.297	0.303	0.307	0.302		1	0.151	1.90	1
leiomyosarcoma	SK-UT-1	Dasatinib & Topotecan	100	0.232	0.283	0.324	0.280		1			
leiomyosarcoma	SK-UT-1	Dasatinib & Topotecan	13.333	0.012	0.002	0.001	0.005		1			
leiomyosarcoma	SK-UT-1	Dasatinib & Topotecan	500	0.398	0.473	0.642	0.504	0.140	2			
leiomyosarcoma	SK-UT-1	Dasatinib & Triciribine	0.1	0.239	0.248	0.254	0.247		1	0.356	5.84	1
leiomyosarcoma	SK-UT-1	Dasatinib & Triciribine	0.2	0.743	0.796	0.834	0.791		1	0.105	3.71	1
leiomyosarcoma	SK-UT-1	Dasatinib & Triciribine	2	0.282	0.264	0.253	0.266		1	0.182	3.82	1
leiomyosarcoma	SK-UT-1	GX15-070 & Dasatinib	0.03	0.785	0.773	0.764	0.774	0.018	3	0.125	1.71	3
leiomyosarcoma	SK-UT-1	GX15-070 & Doxorubicin	2	0.982	0.973	0.967	0.974		1	0.000	0.04	1
leiomyosarcoma	SK-UT-1	GX15-070 & Doxorubicin	20	1.097	1.078	1.065	1.080		1	0.006	0.50	1
leiomyosarcoma	SK-UT-1	GX15-070 & Etoposide	0.2	0.534	0.545	0.553	0.544		1	0.081	2.01	1
leiomyosarcoma	SK-UT-1	GX15-070 & PXD-101	1	0.804	0.815	0.822	0.814	0.037	2	0.110	1.64	2
leiomyosarcoma	SK-UT-1	GX15-070 & Topotecan	1	0.755	0.773	0.786	0.771		1	0.012	0.54	1
leiomyosarcoma	SK-UT-1	GX15-070 & Topotecan	10	1.016	0.984	0.962	0.987		1	0.053	3.46	1
leiomyosarcoma	SK-UT-1	GX15-070 & Vorinostat	0.03	1.196	1.204	1.210	1.204		1	0.031	0.98	1
leiomyosarcoma	SK-UT-1	GX15-070 & Vorinostat	0.06	1.154	1.173	1.187	1.171	0.050	2	0.108	1.46	2
leiomyosarcoma	SK-UT-1	MK-2206 & 4-HC	1	0.956	0.978	0.994	0.976	0.376	2	0.047	2.03	2
leiomyosarcoma	SK-UT-1	MK-2206 & 4-HC & Doxorubicin	1	0.775	0.781	0.786	0.780	0.146	3	0.086	2.27	3
leiomyosarcoma	SK-UT-1	MK-2206 & Cisplatin	0.5	1.533	2.716	4.175	2.580	0.506	3	0.122	4.76	3
leiomyosarcoma	SK-UT-1	MK-2206 & Cisplatin	0.6667	1.216	1.192	1.230	1.213		1	0.114	4.47	1
leiomyosarcoma	SK-UT-1	MK-2206 & Dasatinib	1	0.114	0.462	1.196	0.591		1	0.495	4.08	1
leiomyosarcoma	SK-UT-1	MK-2206 & Doxorubicin	100	0.523	0.580	0.623	0.576		1	0.251	3.65	1
leiomyosarcoma	SK-UT-1	MK-2206 & Doxorubicin	5	0.880	0.872	0.867	0.873		1	0.081	5.55	1
leiomyosarcoma	SK-UT-1	MK-2206 & Doxorubicin	50	0.632	0.614	0.602	0.616	0.078	6	0.134	2.89	6
leiomyosarcoma	SK-UT-1	MK-2206 & Etoposide	5	0.469	0.664	0.921	0.685	0.282	3	0.214	4.10	3
leiomyosarcoma	SK-UT-1	MK-2206 & Etoposide	6.6667	0.242	0.206	0.200	0.216		1	0.230	6.22	1
leiomyosarcoma	SK-UT-1	MK-2206 & MK-8669	0.2	0.581	0.933	1.501	0.913	0.161	12	0.227	4.90	9
leiomyosarcoma	SK-UT-1	MK-2206 & MK-8669	2	0.192	0.652	1.529	0.645	0.281	2	0.191	4.33	2
leiomyosarcoma	SK-UT-1	MK-2206 & Topotecan	50	0.574	0.748	0.925	0.749	0.044	3	0.120	3.53	3
leiomyosarcoma	SK-UT-1	MK-2206 & Topotecan	500	1.596	6.632	19.574	5.917		1	0.138	3.62	1
leiomyosarcoma	SK-UT-1	MK-2206 & Topotecan	66.667	0.449	0.391	0.366	0.402		1	0.184	5.70	1
leiomyosarcoma	SK-UT-1	MK-8669 & MK-2206	0.2	1.009	1.024	1.035	1.022		1	0.103	1.79	1
leiomyosarcoma	SK-UT-1	MK-8669 & MK-2206	5	0.642	0.612	0.671	0.642	0.104	3			
leiomyosarcoma	SK-UT-1	PXD-101 & Doxorubicin	2	0.881	0.876	0.873	0.876	0.032	2	0.105	4.74	1
leiomyosarcoma	SK-UT-1	PXD-101 & Etoposide	0.1	0.780	0.759	0.745	0.761	0.020	2	0.094	3.54	1
leiomyosarcoma	SK-UT-1	PXD-101 & Topotecan	1	0.613	0.593	0.579	0.595	0.052	2	0.083	3.53	1
leiomyosarcoma	SK-UT-1	Rapamycin & Ara-C	10	0.520	0.531	0.538	0.530		1	0.099	2.45	1
leiomyosarcoma	SK-UT-1	Selumetinib & Doxorubicin	0.5	0.789	0.762	0.744	0.765		1	0.024	0.40	1
leiomyosarcoma	SK-UT-1	Selumetinib & Doxorubicin	10	0.951	0.911	0.885	0.916		1	0.111	1.27	1
leiomyosarcoma	SK-UT-1	Selumetinib & Etoposide	0.15	1.007	0.990	0.979	0.992		1	0.024	0.92	1
leiomyosarcoma	SK-UT-1	Selumetinib & Etoposide	0.2	0.802	0.788	0.779	0.790		1	0.069	1.45	1
leiomyosarcoma	SK-UT-1	Selumetinib & Topotecan	2	1.033	0.989	0.960	0.994		1	0.007	0.28	1
leiomyosarcoma	SK-UT-1	Selumetinib & Topotecan	3	0.928	0.923	0.920	0.923		1	0.024	0.71	1

Sarcoma type	Cell line	Drug combination	Molar ratio	CI75	CI90	CI95	CI(mean)	CI(SEM)	n	EOHSA (mean)	neg log p-Value	n
leiomyosarcoma	SK-UT-1	Sunitinib & Ara-C	2	1.561	1.597	1.622	1.593		1	0.072	1.99	1
leiomyosarcoma	SK-UT-1	Vorinostat & Cisplatin	0.2	0.751	0.660	0.615	0.675	0.360	3	0.048	3.66	2
leiomyosarcoma	SK-UT-1	Vorinostat & Etoposide	2	0.729	0.691	0.679	0.700	0.174	3	0.112	5.39	2
leiomyosarcoma	SK-UT-1	Vorinostat & MK-8669	0.05	0.934	0.990	1.033	0.986		1			
leiomyosarcoma	SK-UT-1	Vorinostat & MK-8669	0.5	0.335	1.083	2.414	0.957		1			
leiomyosarcoma	SK-UT-1	Vorinostat & Topotecan	8	0.537	0.377	0.309	0.408	0.067	3	0.079	3.28	2
liposarcoma	SW-872	4-HC & Doxorubicin	50	1.294	1.277	1.266	1.279	0.199	2	0.020	0.94	2
liposarcoma	SW-872	ABT-888 & Cisplatin	5	1.168	1.215	1.248	1.210		1	-0.010	0.08	1
liposarcoma	SW-872	ABT-888 & Gemcitabine	250	1.052	1.060	1.066	1.059		1	0.023	0.65	1
liposarcoma	SW-872	ABT-888 & Triciribine	2.5	0.781	0.774	0.769	0.775		1	0.069	1.39	1
liposarcoma	SW-872	Dasatinib & Ara-C	1	0.387	0.343	0.316	0.349		1	0.312	5.81	1
liposarcoma	SW-872	Dasatinib & Ara-C	5	0.172	0.167	0.163	0.167	0.020	2	0.335	4.87	2
liposarcoma	SW-872	Dasatinib & Doxorubicin	1	0.170	0.149	0.136	0.152		1	0.237	5.38	1
liposarcoma	SW-872	Dasatinib & Doxorubicin	5	0.329	0.303	0.286	0.306		1	0.210	3.48	1
liposarcoma	SW-872	Dasatinib & Etoposide	0.2	0.254	0.505	1.076	0.612		1			
liposarcoma	SW-872	Dasatinib & Etoposide	1	0.449	0.462	0.503	0.471	0.052	4	0.194	3.56	2
liposarcoma	SW-872	Dasatinib & Gemcitabine	10	0.214	0.224	0.231	0.223	0.032	2	0.185	2.52	2
liposarcoma	SW-872	Dasatinib & MK-2206	0.1	0.258	0.304	0.370	0.310		1	0.201	2.59	1
liposarcoma	SW-872	Dasatinib & MK-2206	0.2	0.397	0.513	0.642	0.517	0.352	3	0.190	3.03	3
liposarcoma	SW-872	Dasatinib & MK-2206	0.5	0.291	0.414	0.588	0.431		1	0.245	5.68	1
liposarcoma	SW-872	Dasatinib & MK-2206	1	0.200	0.542	1.135	0.544	0.241	4	0.151	3.96	4
liposarcoma	SW-872	Dasatinib & MK-2206	2.5	0.169	0.258	0.344	0.257		1	0.165	5.61	1
liposarcoma	SW-872	Dasatinib & Topotecan	1.3333	0.132	0.144	0.170	0.149		1			
liposarcoma	SW-872	Dasatinib & Topotecan	10	0.355	0.336	0.324	0.339	0.045	2	0.190	3.54	2
liposarcoma	SW-872	Dasatinib & Topotecan	100	0.540	0.668	0.836	0.681	0.070	3			
liposarcoma	SW-872	Dasatinib & Topotecan	13.333	0.127	0.012	0.004	0.048		1			
liposarcoma	SW-872	Dasatinib & Topotecan	500	0.311	0.118	0.067	0.165	0.076	2			
liposarcoma	SW-872	Dasatinib & Triciribine	0.1	0.443	0.446	0.448	0.446		1	0.252	3.96	1
liposarcoma	SW-872	Dasatinib & Triciribine	0.2	0.613	0.626	0.635	0.625		1	0.158	3.59	1
liposarcoma	SW-872	Dasatinib & Triciribine	0.5	0.102	0.099	0.097	0.099	0.039	2	0.368	6.44	2
liposarcoma	SW-872	GX15-070 & Dasatinib	0.03	1.073	1.055	1.042	1.057	0.149	3	0.083	1.80	3
liposarcoma	SW-872	GX15-070 & Doxorubicin	2	1.389	1.365	1.350	1.368		1	0.022	1.47	1
liposarcoma	SW-872	GX15-070 & Etoposide	0.02	1.063	1.046	1.035	1.048		1	-0.003	0.05	1
liposarcoma	SW-872	GX15-070 & PXD-101	1	1.116	1.167	1.205	1.163		1	0.072	1.70	1
liposarcoma	SW-872	GX15-070 & Topotecan	1	1.203	1.191	1.182	1.192		1	-0.010	0.47	1
liposarcoma	SW-872	GX15-070 & Vorinostat	0.06	1.871	1.912	1.940	1.908	0.016	2	0.022	0.36	2
liposarcoma	SW-872	MK-2206 & 4-HC	1	0.895	0.890	0.888	0.891	0.077	2	0.065	1.93	2
liposarcoma	SW-872	MK-2206 & 4-HC & Doxorubicin	1	0.833	0.829	0.826	0.829	0.128	3	0.077	2.37	3
liposarcoma	SW-872	MK-2206 & Cisplatin	0.5	1.380	1.384	1.390	1.385	0.140	3	-0.010	0.30	3
liposarcoma	SW-872	MK-2206 & Cisplatin	0.6667	2.015	2.155	2.281	2.147		1	0.036	1.19	1
liposarcoma	SW-872	MK-2206 & Dasatinib	1	0.256	0.342	0.416	0.338		1	0.368	4.15	1
liposarcoma	SW-872	MK-2206 & Doxorubicin	100	0.736	0.764	0.785	0.762		1	0.186	3.55	1
liposarcoma	SW-872	MK-2206 & Doxorubicin	5	0.738	0.740	0.742	0.740		1	0.104	4.34	1
liposarcoma	SW-872	MK-2206 & Doxorubicin	50	0.724	0.693	0.675	0.697	0.101	6	0.146	2.59	6
liposarcoma	SW-872	MK-2206 & Etoposide	5	0.582	0.628	0.670	0.627	0.035	2	0.179	3.00	2
liposarcoma	SW-872	MK-2206 & MK-8669	0.2	0.862	1.240	2.042	1.213	0.105	10	0.221	4.84	7
liposarcoma	SW-872	MK-2206 & MK-8669	2	0.189	0.357	0.552	0.366		1	0.183	2.63	1
liposarcoma	SW-872	MK-2206 & Topotecan	50	0.509	0.510	0.520	0.513	0.066	3	0.104	1.82	3
liposarcoma	SW-872	MK-2206 & Topotecan	500	0.486	0.756	1.023	0.755		1	0.128	1.80	1
liposarcoma	SW-872	MK-8669 & MK-2206	5	1.164	1.669	2.744	1.631	0.319	3			
liposarcoma	SW-872	PXD-101 & Doxorubicin	2	0.827	0.795	0.774	0.799		1			
liposarcoma	SW-872	PXD-101 & Etoposide	0.1	0.768	0.736	0.716	0.740		1			
liposarcoma	SW-872	PXD-101 & Topotecan	1	0.884	0.863	0.850	0.866		1			
liposarcoma	SW-872	Rapamycin & Sunitinib	10	0.740	0.846	0.926	0.837		1	0.045	0.75	1
liposarcoma	SW-872	Saracatinib & Ara-C	10	0.288	0.280	0.275	0.281		1	0.303	5.29	1
liposarcoma	SW-872	Saracatinib & Gemcitabine	100	0.696	0.689	0.684	0.690		1	0.125	2.35	1
liposarcoma	SW-872	Selumetinib & Doxorubicin	0.5	0.833	0.823	0.817	0.824		1	0.041	2.20	1
liposarcoma	SW-872	Selumetinib & Doxorubicin	10	1.075	1.016	0.978	1.023		1	0.139	1.78	1
liposarcoma	SW-872	Selumetinib & Etoposide	0.15	0.922	0.906	0.896	0.908		1	0.101	4.57	1
liposarcoma	SW-872	Selumetinib & Topotecan	2	1.128	1.113	1.103	1.115		1	0.029	1.39	1
liposarcoma	SW-872	Selumetinib & Topotecan	3	1.076	1.052	1.036	1.055		1	0.013	0.70	1

Sarcoma type	Cell line	Drug combination	Molar ratio	CI75	CI90	CI95	CI(mean)	CI(SEM)	n	EOHSA (mean)	neg log p-Value	n
liposarcoma	SW-872	Sunitinib & Ara-C	1	1.083	1.024	0.986	1.031		1	0.008	0.17	1
liposarcoma	SW-872	Sunitinib & Ara-C	2	1.821	1.765	1.728	1.772		1	0.009	0.07	1
liposarcoma	SW-872	Vorinostat & Cisplatin	0.2	0.746	0.642	0.585	0.658	0.243	4	0.068	2.16	3
liposarcoma	SW-872	Vorinostat & Etoposide	2	0.532	0.435	0.384	0.450	0.152	3	0.185	3.00	2
liposarcoma	SW-872	Vorinostat & MK-8669	0.05	1.170	1.136	1.115	1.140	0.570	1			
liposarcoma	SW-872	Vorinostat & Topotecan	8	0.711	0.710	0.717	0.713	0.131	4	0.053	1.41	3
fibrosarcoma	HT-1080	Dasatinib & Ara-C	1	0.629	0.620	0.614	0.621	0.264	2	0.115	3.04	2
fibrosarcoma	HT-1080	Dasatinib & Ara-C	5	0.383	0.370	0.362	0.372	0.074	3	0.244	4.98	3
fibrosarcoma	HT-1080	Dasatinib & Ara-C	50	0.373	0.360	0.351	0.361		1	0.202	3.26	1
fibrosarcoma	HT-1080	Dasatinib & Gemcitabine	50	0.872	0.859	0.851	0.860		1	0.073	1.35	1
fibrosarcoma	HT-1080	Dasatinib & MK-2206	0.2	1.464	2.250	3.087	2.193	1.132	3	0.082	1.83	3
fibrosarcoma	HT-1080	Dasatinib & MK-2206	1	0.389	0.554	0.764	0.569	0.173	3	0.182	2.87	3
fibrosarcoma	HT-1080	Dasatinib & Triciribine	0.1	0.413	0.427	0.437	0.426		1	0.297	3.39	1
fibrosarcoma	HT-1080	Dasatinib & Triciribine	0.125	0.212	0.210	0.209	0.210	0.127	2	0.271	6.39	2
fibrosarcoma	HT-1080	Dasatinib & Triciribine	0.25	0.121	0.116	0.113	0.117	0.067	2	0.359	7.83	2
fibrosarcoma	HT-1080	Dasatinib & Triciribine	0.5	0.326	0.340	0.350	0.339		1	0.240	3.74	1
fibrosarcoma	HT-1080	Dasatinib & Triciribine	1	0.140	0.133	0.128	0.133		1	0.279	3.64	1
fibrosarcoma	HT-1080	GX15-070 & Dasatinib	0.03	0.780	0.771	0.765	0.772	0.002	2	0.128	3.35	2
fibrosarcoma	HT-1080	GX15-070 & Doxorubicin	2	1.667	1.649	1.637	1.651		1	-0.010	0.13	1
fibrosarcoma	HT-1080	GX15-070 & Doxorubicin	20	0.992	1.034	1.066	1.031	0.048	2	0.119	2.60	2
fibrosarcoma	HT-1080	GX15-070 & Etoposide	0.02	1.041	1.040	1.040	1.041		1	0.017	0.59	1
fibrosarcoma	HT-1080	GX15-070 & Etoposide	0.2	0.805	0.755	0.728	0.763	0.184	3	0.158	1.84	3
fibrosarcoma	HT-1080	GX15-070 & Etoposide	1	1.042	1.025	1.014	1.027		1	0.006	0.17	1
fibrosarcoma	HT-1080	GX15-070 & Topotecan	1	1.261	1.293	1.315	1.290		1	0.016	0.63	1
fibrosarcoma	HT-1080	GX15-070 & Topotecan	10	1.103	1.140	1.167	1.137	0.317	2	0.073	2.37	2
fibrosarcoma	HT-1080	MK-2206 & Cisplatin	0.5	0.634	0.661	0.682	0.659	0.019	2	0.140	2.86	2
fibrosarcoma	HT-1080	MK-2206 & Doxorubicin	100	0.786	0.725	0.697	0.736		1	0.219	5.53	1
fibrosarcoma	HT-1080	MK-2206 & Doxorubicin	20	0.585	0.590	0.598	0.591	0.114	4	0.260	3.16	4
fibrosarcoma	HT-1080	MK-2206 & Doxorubicin	25	0.834	0.853	0.868	0.852		1	0.035	1.20	1
fibrosarcoma	HT-1080	MK-2206 & Doxorubicin	50	0.413	0.436	0.453	0.434	0.013	2	0.202	5.60	2
fibrosarcoma	HT-1080	MK-2206 & Etoposide	1	0.746	0.782	0.817	0.782	0.138	4	0.161	3.14	4
fibrosarcoma	HT-1080	MK-2206 & Etoposide	5	0.178	0.149	0.135	0.154	0.077	2	0.168	3.11	2
fibrosarcoma	HT-1080	MK-2206 & MK-8669	0.2	0.822	0.842	0.871	0.845	0.124	9	0.321	3.30	8
fibrosarcoma	HT-1080	MK-2206 & MK-8669	2	0.288	0.309	0.325	0.307		1			
fibrosarcoma	HT-1080	MK-2206 & Topotecan	50	0.034	0.010	0.005	0.016		1	0.097	1.86	1
fibrosarcoma	HT-1080	PXD-101 & Doxorubicin	2	0.619	0.635	0.645	0.633		1	0.094	1.91	1
fibrosarcoma	HT-1080	PXD-101 & Etoposide	0.1	0.709	0.692	0.681	0.694	0.198	2	0.095	1.62	2
fibrosarcoma	HT-1080	PXD-101 & Topotecan	0.3333							0.138	2.83	1
fibrosarcoma	HT-1080	PXD-101 & Topotecan	1	1.072	1.070	1.069	1.070	0.677	2	0.044	1.87	2
fibrosarcoma	HT-1080	Saracatinib & Ara-C	10	0.742	0.767	0.785	0.765		1	0.082	1.27	1
fibrosarcoma	HT-1080	Selumetinib & Doxorubicin	0.5	1.092	1.044	1.013	1.049		1	0.014	0.18	1
fibrosarcoma	HT-1080	Selumetinib & Etoposide	0.15	0.287	0.273	0.264	0.275		1	0.113	2.46	1
fibrosarcoma	HT-1080	Sunitinib & Ara-C	2	3.288	3.091	2.964	3.111		1	-0.209	1.34	1
fibrosarcoma	HT-1080	Triciribine & Dasatinib	0.5	0.157	0.128	0.111	0.132		1	0.317	6.25	1
fibrosarcoma	HT-1080	Triciribine & Dasatinib	5	0.050	0.022	0.014	0.029		1	0.321	4.31	1
fibrosarcoma	HT-1080	Triciribine & Doxorubicin	800	0.575	0.557	0.545	0.559		1	0.140	3.62	1
fibrosarcoma	HT-1080	Triciribine & Topotecan	400	0.666	0.656	0.649	0.657		1	0.098	3.50	1
fibrosarcoma	HT-1080	Vorinostat & Cisplatin	0.2	1.837	2.788	3.808	2.668	2.450	5	0.149	2.81	5
fibrosarcoma	HT-1080	Vorinostat & Etoposide	2	0.230	0.183	0.163	0.192	0.038	5	0.240	2.79	5
fibrosarcoma	HT-1080	Vorinostat & MK-8669	0.05	0.960	0.998	1.037	0.999	0.218	2	0.151	2.00	2
fibrosarcoma	HT-1080	Vorinostat & Topotecan	8	0.223	0.194	0.195	0.204	0.034	5	0.261	3.50	5

Supplemental Table 2S. Sequential drug combination effect summary. The sarcoma cell line and molar drug ratio for each drug combination is indicated. Time1 indicates first drug incubation time (hours) for first drug listed in combination. Time2 indicates incubation time (hours) for secondary drug(s) added. Combination index (CI) values for effect levels of 0.75, 0.9 and 0.95 were calculated for each independent experiment by the method of Chou and Talalay and the mean CI value "CI(mean)" was calculated using the CI for each effect level. The standard error of the mean for CI values across independent experiments are shown "CI(SEM)". EoHSA values and level of significance are represented in EoHSA(mean) and –log p-Value columns, respectively. Each line represents the calculated mean value for the number of independent experiments (n).

Sarcoma type	Cell line	Drug combination	Molar ratio	Time1	Time2	CI75	CI90	CI95	CI(mean)	CI(SEM)	n	EOHSA (mean)	neg log p-Value	n
Ewing	A-673	Ara-C & Dasatinib	0.25	24	48	1.682	1.682	1.682	1.682		1	0.002	0.36	1
Ewing	A-673	Dasatinib & Ara-C	0.25	24	48	0.117	0.110	0.105	0.111		1	0.148	9.52	1
Ewing	A-673	Dasatinib & Triciribine	1	24	48	0.263	0.257	0.253	0.258		1	0.313	4.19	1
Ewing	A-673	GX15-070 & Dasatinib	0.02	24	48	1.037	1.026	1.020	1.028	0.061	3	0.110	2.95	3
Ewing	A-673	GX15-070 & PXD-101	0.1	24	48	1.294	1.290	1.287	1.290	0.004	3	0.042	0.95	3
Ewing	A-673	GX15-070 & Vorinostat	0.02	24	48	1.222	1.200	1.185	1.202	0.092	3	0.045	1.24	3
Ewing	A-673	PXD-101 & Doxorubicin	1	24	48	1.938	1.953	1.964	1.952	0.083	2	-0.008	0.50	2
Ewing	A-673	PXD-101 & Etoposide	0.05	24	48	1.656	1.651	1.649	1.652	0.201	3	-0.027	0.76	3
Ewing	A-673	PXD-101 & Etoposide	3.33E-02	24	48	0.420	0.415	0.412	0.416		1	0.170	4.22	1
Ewing	A-673	PXD-101 & Topotecan	0.33333	24	48	0.577	0.588	0.595	0.587		1	0.143	4.48	1
Ewing	A-673	PXD-101 & Topotecan	2.5	24	48	1.145	1.058	1.003	1.068		1	0.021	0.10	1
Ewing	A-673	Triciribine & Dasatinib	1	24	48	0.200	0.195	0.192	0.196	0.056	2	0.314	4.22	2
Ewing	RD-ES	Ara-C & Dasatinib	0.25	24	48	0.491	0.475	0.466	0.478		1	0.232	4.22	1
Ewing	RD-ES	GX15-070 & Dasatinib	0.01	24	48	1.655	1.558	1.496	1.570		1	0.020	0.32	1
Ewing	RD-ES	GX15-070 & Dasatinib	0.02	24	48	0.560	0.494	0.453	0.502		1	0.255	3.01	1
Ewing	RD-ES	GX15-070 & Doxorubicin	1	24	48	0.667	0.677	0.684	0.676		1	0.028	2.01	1
Ewing	RD-ES	GX15-070 & Etoposide	0.05	24	48	1.470	1.445	1.429	1.448		1	0.004	0.37	1
Ewing	RD-ES	GX15-070 & PXD-101	0.1	24	48	1.756	1.760	1.763	1.760	0.033	2	-0.050	1.19	2
Ewing	RD-ES	GX15-070 & Vorinostat	0.02	24	48	0.976	0.950	0.932	0.953		1	0.086	5.23	1
Ewing	RD-ES	PXD-101 & Doxorubicin	0.33333	24	48	0.752	0.591	0.513	0.618		1	0.175	3.84	1
Ewing	RD-ES	PXD-101 & Doxorubicin	1	24	48	0.932	0.907	0.890	0.909	0.098	2	0.111	4.37	2
Ewing	RD-ES	PXD-101 & Etoposide	0.05	24	48	1.166	1.151	1.141	1.153		1	0.029	1.78	1
Ewing	RD-ES	Selumetinib & Doxorubicin	1	24	48	1.807	1.830	1.847	1.828		1	0.038	0.65	1
Ewing	SK-ES-1	Ara-C & Dasatinib	0.25	24	48	0.881	0.832	0.800	0.838		1	0.034	1.69	1
Ewing	SK-ES-1	Dasatinib & Ara-C	0.25	24	48	0.015	0.018	0.021	0.018		1	0.085	3.99	1
Ewing	SK-ES-1	Dasatinib & Etoposide	0.4	48	24	0.485	0.675	0.851	0.670		1			
Ewing	SK-ES-1	Dasatinib & Topotecan	0.66667	48	24	0.193	1.449	7.901	1.303		1			
Ewing	SK-ES-1	Dasatinib & Topotecan	2.66667	48	24	0.343	1.733	5.525	1.487		1			
Ewing	SK-ES-1	Dasatinib & Triciribine	1	24	48	0.390	0.380	0.373	0.381	0.011	2	0.219	4.74	2
Ewing	SK-ES-1	GX15-070 & Dasatinib	0.02	24	48	0.913	0.899	0.891	0.901	0.079	3	0.074	2.58	3
Ewing	SK-ES-1	GX15-070 & PXD-101	0.1	24	48	1.245	1.284	1.312	1.280	0.194	3	-0.010	0.82	3
Ewing	SK-ES-1	GX15-070 & Vorinostat	0.02	24	48	1.169	1.172	1.175	1.172	0.073	2	0.009	0.41	2
Ewing	SK-ES-1	MK-2206 & Doxorubicin	20	24	48	0.782	0.925	1.064	0.924	0.087	3	0.082	2.17	3
Ewing	SK-ES-1	MK-2206 & Etoposide	1	24	48	0.446	0.629	0.865	0.647	0.038	2	0.086	1.79	2
Ewing	SK-ES-1	MK-2206 & MK-8669	0.2	24	48	0.815	0.947	1.061	0.941	0.079	3	0.171	3.08	3
Ewing	SK-ES-1	MK-8669 & Vorinostat	2	24	48	0.205	1.155	4.072	0.988		1			
Ewing	SK-ES-1	PXD-101 & Doxorubicin	1	24	48	0.730	0.716	0.707	0.718		1	0.129	2.37	1
Ewing	SK-ES-1	Sorafenib & Topotecan	10	24	48	1.667	2.252	2.925	2.223		1	0.008	0.38	1
Ewing	SK-ES-1	Topotecan & Sorafenib	0.002	24	48	7.426	20.198	41.778	18.231	17.087	3	0.036	2.08	2
Ewing	SK-ES-1	Triciribine & Dasatinib	1	24	48	0.553	0.529	0.515	0.532	0.056	2	0.150	4.96	2
Ewing	SK-ES-1	Vorinostat & Cisplatin	0.2	48	24	1.049	0.880	0.814	0.914	0.102	2	0.005	1.52	2
Ewing	SK-ES-1	Vorinostat & Etoposide	0.2	48	24	0.813	0.941	1.207	0.987	0.262	2	0.075	1.87	2
Ewing	SK-ES-1	Vorinostat & MK-8669	0.05	24	48	1.098	1.233	1.340	1.224		1			
Ewing	SK-ES-1	Vorinostat & Topotecan	0.4	48	24	0.418	0.630	0.867	0.638		1	0.079	3.53	1
osteosarcoma	MNNG HOS	Ara-C & Dasatinib	0.25	24	48	0.274	0.270	0.267	0.271		1	0.309	5.05	1
osteosarcoma	MNNG HOS	Dasatinib & Ara-C	0.25	24	48	0.109	0.086	0.073	0.089		1	0.184	7.01	1
osteosarcoma	MNNG HOS	Dasatinib & Etoposide	0.4	48	24	0.260	0.354	0.437	0.350		1			
osteosarcoma	MNNG HOS	Dasatinib & Topotecan	0.66667	48	24	0.231	0.168	0.135	0.178		1			
osteosarcoma	MNNG HOS	Dasatinib & Topotecan	2.66667	48	24	0.281	0.232	0.204	0.239		1			
osteosarcoma	MNNG HOS	GX15-070 & Dasatinib	0.02	24	48	1.108	1.121	1.132	1.120	0.171	3	0.096	2.84	3
osteosarcoma	MNNG HOS	GX15-070 & PXD-101	0.1	24	48	1.792	1.887	1.955	1.878		1	0.024	0.43	1
osteosarcoma	MNNG HOS	GX15-070 & Vorinostat	0.02	24	48	1.438	1.378	1.340	1.385	0.276	3	0.054	2.72	3
osteosarcoma	MNNG HOS	MK-2206 & Doxorubicin	20	24	48	0.715	0.657	0.630	0.667	0.031	2	0.164	4.21	2
osteosarcoma	MNNG HOS	MK-2206 & Etoposide	1	24	48	0.707	0.674	0.659	0.680	0.078	3	0.167	3.92	3
osteosarcoma	MNNG HOS	MK-2206 & MK-8669	0.2	24	48	1.176	1.202	1.221	1.200	0.083	3	0.187	3.22	3
osteosarcoma	MNNG HOS	MK-8669 & Vorinostat	2	24	48	0.552	0.748	1.000	0.767	0.211	4	0.343	5.38	2
osteosarcoma	MNNG HOS	PXD-101 & Doxorubicin	0.1	24	48	0.754	0.726	0.709	0.730		1	0.099	4.18	1
osteosarcoma	MNNG HOS	PXD-101 & Doxorubicin	0.33333	24	48	0.952	0.908	0.880	0.913		1	0.068	1.32	1
osteosarcoma	MNNG HOS	PXD-101 & Doxorubicin	1	24	48	1.382	1.385	1.388	1.385		1	0.007	0.14	1
osteosarcoma	MNNG HOS	PXD-101 & Etoposide	0.05	24	48	1.469	1.591	1.683	1.578	0.495	2	0.028	1.26	2

Sarcoma type	Cell line	Drug combination	Molar ratio	Time1	Time2	CI75	CI90	CI95	CI(mean)	CI(SEM)	n	EOHSA (mean)	neg log p-Value	n
osteosarcoma	MNNG HOS	PXD-101 & Etoposide	3.33E-02	24	48	0.877	0.868	0.861	0.869		1	0.101	4.39	1
osteosarcoma	MNNG HOS	PXD-101 & Topotecan	0.25	24	48	1.042	1.064	1.079	1.062		1	0.045	1.78	1
osteosarcoma	MNNG HOS	PXD-101 & Topotecan	0.33333	24	48	0.843	0.850	0.855	0.849		1	0.061	2.83	1
osteosarcoma	MNNG HOS	PXD-101 & Topotecan	2.5	24	48	0.718	0.718	0.718	0.718		1	0.193	3.57	1
osteosarcoma	MNNG HOS	Sorafenib & Topotecan	10	24	48	1.412	1.776	2.188	1.763	0.460	2	0.008	0.38	1
osteosarcoma	MNNG HOS	Topotecan & Sorafenib	0.002	24	48	2.979	5.022	7.628	4.755	3.029	3	0.002	2.11	2
osteosarcoma	MNNG HOS	Vorinostat & Cisplatin	0.2	48	24	3.252	11.701	28.268	10.246		1	0.238	7.82	1
osteosarcoma	MNNG HOS	Vorinostat & Etoposide	0.2	48	24	0.631	0.921	1.344	0.909	0.836	2	0.050	1.65	2
osteosarcoma	MNNG HOS	Vorinostat & MK-8669	0.05	24	48	0.815	0.815	0.833	0.821	0.046	4	0.177	3.67	2
osteosarcoma	MNNG HOS	Vorinostat & Topotecan	0.4	48	24	0.502	0.583	0.693	0.593	0.259	2	0.087	3.28	2
osteosarcoma	U2-OS	Ara-C & Dasatinib	0.25	24	48	0.246	0.215	0.197	0.219		1	0.337	3.29	1
osteosarcoma	U2-OS	GX15-070 & Dasatinib	0.01	24	48	1.168	1.165	1.163	1.166		1	0.098	1.82	1
osteosarcoma	U2-OS	GX15-070 & Dasatinib	0.02	24	48	0.944	0.920	0.904	0.923	0.172	2	0.214	2.18	2
osteosarcoma	U2-OS	GX15-070 & PXD-101	0.1	24	48	1.573	1.704	1.805	1.689	0.321	2	0.033	0.44	2
osteosarcoma	U2-OS	GX15-070 & Vorinostat	0.01	24	48	2.139	2.371	2.542	2.345		1	0.012	0.12	1
osteosarcoma	U2-OS	GX15-070 & Vorinostat	0.02	24	48	1.406	1.390	1.380	1.392	0.233	2	0.025	0.38	2
osteosarcoma	U2-OS	MK-8669 & Vorinostat	2	24	48	0.630	0.826	1.089	0.848	0.320	2	0.169	4.50	2
osteosarcoma	U2-OS	PXD-101 & Doxorubicin	0.33333	24	48	0.801	0.809	0.814	0.808		1	0.127	4.11	1
osteosarcoma	U2-OS	PXD-101 & Doxorubicin	1	24	48	1.180	1.188	1.193	1.187	0.072	2	0.119	5.87	2
osteosarcoma	U2-OS	PXD-101 & Etoposide	0.025	24	48	1.088	1.079	1.073	1.080		1	0.124	4.22	1
osteosarcoma	U2-OS	PXD-101 & Etoposide	0.05	24	48	1.191	1.217	1.236	1.215	0.090	3	0.047	2.51	3
osteosarcoma	U2-OS	PXD-101 & Etoposide	3.33E-02	24	48	1.069	1.071	1.073	1.071		1	0.034	1.53	1
osteosarcoma	U2-OS	PXD-101 & Topotecan	0.025	24	48	0.394	0.388	0.384	0.389		1	0.254	3.18	1
osteosarcoma	U2-OS	Selumetinib & Doxorubicin	5	24	48	1.325	1.305	1.291	1.307		1	-0.009	0.25	1
osteosarcoma	U2-OS	Selumetinib & Topotecan	5	24	48	1.229	1.250	1.264	1.248		1	-0.005	0.13	1
osteosarcoma	U2-OS	Topotecan & Sorafenib	0.002	24	48	3.755	5.995	8.241	5.704		1	0.030	1.87	1
osteosarcoma	U2-OS	Vorinostat & Cisplatin	0.2	48	24	0.555	0.462	0.411	0.476		1	0.100	4.63	1
osteosarcoma	U2-OS	Vorinostat & Etoposide	0.2	48	24	0.881	0.799	0.759	0.813	0.092	2	0.065	3.31	2
osteosarcoma	U2-OS	Vorinostat & MK-8669	0.05	24	48	0.921	0.788	0.732	0.814	0.003	2	0.105	3.13	2
osteosarcoma	U2-OS	Vorinostat & Topotecan	0.4	48	24	0.533	0.419	0.365	0.439	0.019	2	0.104	2.52	2
rhabdomyosarcoma	A-204	Ara-C & Dasatinib	0.25	24	48	0.235	0.221	0.214	0.223		1	0.393	4.93	1
rhabdomyosarcoma	A-204	GX15-070 & Dasatinib	0.01	24	48	0.550	0.506	0.478	0.511		1	0.142	2.98	1
rhabdomyosarcoma	A-204	GX15-070 & Dasatinib	0.02	24	48	0.392	0.386	0.381	0.386	0.023	3	0.291	5.95	3
rhabdomyosarcoma	A-204	GX15-070 & PXD-101	0.1	24	48	0.947	0.983	1.015	0.982	0.237	4	0.121	2.24	4
rhabdomyosarcoma	A-204	GX15-070 & Vorinostat	0.01	24	48	0.929	0.900	0.881	0.903		1	0.047	2.07	1
rhabdomyosarcoma	A-204	GX15-070 & Vorinostat	0.02	24	48	0.845	0.816	0.797	0.819	0.162	3	0.136	3.29	3
rhabdomyosarcoma	A-204	MK-8669 & Vorinostat	2	24	48	0.554	0.923	1.449	0.887	0.335	2	0.200	4.39	2
rhabdomyosarcoma	A-204	PXD-101 & Doxorubicin	0.33333	24	48	1.054	1.089	1.114	1.086		1	0.122	2.83	1
rhabdomyosarcoma	A-204	PXD-101 & Doxorubicin	1	24	48	1.447	1.372	1.323	1.381		1	0.080	4.93	1
rhabdomyosarcoma	A-204	PXD-101 & Etoposide	0.025	24	48	1.016	1.000	0.989	1.002		1	0.052	0.94	1
rhabdomyosarcoma	A-204	PXD-101 & Etoposide	0.05	24	48	1.033	1.013	1.000	1.016	0.127	2	0.073	4.29	2
rhabdomyosarcoma	A-204	PXD-101 & Etoposide	3.33E-02	24	48	1.084	1.064	1.051	1.067		1	0.010	0.28	1
rhabdomyosarcoma	A-204	PXD-101 & Topotecan	0.33333	24	48	0.874	0.849	0.835	0.853		1	0.049	1.37	1
rhabdomyosarcoma	A-204	PXD-101 & Topotecan	2.5	24	48	1.005	0.971	0.949	0.975		1	0.077	4.53	1
rhabdomyosarcoma	A-204	Selumetinib & Doxorubicin	1	24	48	1.287	1.215	1.169	1.224		1	0.178	3.65	1
rhabdomyosarcoma	A-204	Sorafenib & Topotecan	10	24	48	0.312	0.384	0.445	0.380		1	0.183	5.46	1
rhabdomyosarcoma	A-204	Topotecan & Sorafenib	0.002	24	48	0.498	0.541	0.573	0.537		1	0.296	3.72	1
rhabdomyosarcoma	A-204	Vorinostat & Cisplatin	0.2	48	24	0.811	0.961	1.214	0.962	0.717	2	0.044	2.45	2
rhabdomyosarcoma	A-204	Vorinostat & Etoposide	0.2	48	24	0.760	0.854	1.060	0.870	0.645	2	0.021	0.39	2
rhabdomyosarcoma	A-204	Vorinostat & MK-8669	0.05	24	48	0.750	0.680	0.648	0.693	0.058	2	0.129	3.34	2
rhabdomyosarcoma	A-204	Vorinostat & Topotecan	0.4	48	24	0.904	1.789	3.681	1.813		1	0.039	1.40	1
leiomyosarcoma	SK-LMS-1	Ara-C & Dasatinib	0.25	24	48	0.408	0.415	0.419	0.414		1	0.285	4.66	1
leiomyosarcoma	SK-LMS-1	GX15-070 & Dasatinib	0.01	24	48	0.921	0.894	0.876	0.897		1	0.115	1.61	1
leiomyosarcoma	SK-LMS-1	GX15-070 & Dasatinib	0.02	24	48	0.521	0.517	0.515	0.518	0.203	3	0.273	3.93	3
leiomyosarcoma	SK-LMS-1	GX15-070 & PXD-101	0.1	24	48	0.963	0.968	0.972	0.968		1	0.039	0.59	1
leiomyosarcoma	SK-LMS-1	GX15-070 & Topotecan	0.5	24	48	2.273	2.245	2.226	2.248		1	-0.032	0.75	1
leiomyosarcoma	SK-LMS-1	GX15-070 & Vorinostat	0.01	24	48	1.117	1.121	1.124	1.120		1	0.085	1.13	1
leiomyosarcoma	SK-LMS-1	GX15-070 & Vorinostat	0.02	24	48	1.186	1.216	1.237	1.213		1	0.057	1.01	1
leiomyosarcoma	SK-LMS-1	MK-8669 & Vorinostat	2	24	48	0.507	0.766	1.067	0.780		1	0.106	1.71	1
leiomyosarcoma	SK-LMS-1	PXD-101 & Doxorubicin	0.33333	24	48	2.230	2.285	2.323	2.279		1	-0.016	0.22	1
leiomyosarcoma	SK-LMS-1	PXD-101 & Doxorubicin	1	24	48	0.891	0.817	0.770	0.826		1	0.070	1.76	1
leiomyosarcoma	SK-LMS-1	PXD-101 & Etoposide	0.05	24	48	1.437	1.492	1.534	1.486	0.569	2	0.037	1.30	2
leiomyosarcoma	SK-LMS-1	PXD-101 & Etoposide	3.33E-02	24	48	1.785	1.804	1.818	1.802		1	-0.033	0.49	1
leiomyosarcoma	SK-LMS-1	PXD-101 & Topotecan	2.5	24	48	0.944	1.041	1.113	1.033		1	0.047	2.48	1
leiomyosarcoma	SK-LMS-1	Selumetinib & Doxorubicin	1	24	48	1.049	1.036	1.028	1.038	0.050	2	0.107	2.53	2
leiomyosarcoma	SK-LMS-1	Selumetinib & Doxorubicin	5	24	48	1.072	1.104	1.126	1.101		1	0.015	0.88	1
leiomyosarcoma	SK-LMS-1	Selumetinib & Saracatinib	5	24	48	0.610	0.617	0.621	0.616		1	0.059	0.89	1

Sarcoma type	Cell line	Drug combination	Molar ratio	Time1	Time2	CI75	CI90	CI95	CI(mean)	CI(SEM)	n	EOHSA (mean)	neg log p-Value	n
leiomyosarcoma	SK-LMS-1	Selumetinib & Topotecan	1	24	48	1.206	1.192	1.183	1.194	0.000	2	0.054	0.52	2
leiomyosarcoma	SK-LMS-1	Topotecan & Sorafenib	0.002	24	48	19.864	57.041	117.565	50.946	50.138	2	0.014	0.66	2
leiomyosarcoma	SK-LMS-1	Vorinostat & Cisplatin	0.2	48	24	0.909	0.766	0.694	0.790		1	0.013	0.33	1
leiomyosarcoma	SK-LMS-1	Vorinostat & Etoposide	0.2	48	24	3.017	11.894	30.281	10.280		1	0.247	4.66	1
leiomyosarcoma	SK-LMS-1	Vorinostat & MK-8669	0.05	24	48	0.920	0.832	0.784	0.845	0.071	2	0.078	1.51	2
leiomyosarcoma	SK-LMS-1	Vorinostat & Topotecan	0.4	48	24	0.391	0.301	0.285	0.326		1	-0.120	1.24	1
leiomyosarcoma	SK-UT-1	Ara-C & Dasatinib	0.25	24	48	0.639	0.634	0.631	0.634		1	0.114	2.75	1
leiomyosarcoma	SK-UT-1	Dasatinib & Ara-C	0.25	24	48	0.048	0.046	0.044	0.046		1	0.287	10.29	1
leiomyosarcoma	SK-UT-1	Dasatinib & Etoposide	0.2	48	24	0.376	0.281	0.234	0.297		1			
leiomyosarcoma	SK-UT-1	Dasatinib & Etoposide	0.4	48	24	0.181	0.208	0.234	0.208		1			
leiomyosarcoma	SK-UT-1	Dasatinib & Topotecan	0.66667	48	24	0.400	0.319	0.276	0.332	0.201	2			
leiomyosarcoma	SK-UT-1	Dasatinib & Topotecan	2.66667	48	24	0.575	0.536	0.521	0.544	0.387	2			
leiomyosarcoma	SK-UT-1	GX15-070 & Dasatinib	0.02	24	48	0.891	0.884	0.880	0.885	0.070	3	0.122	3.27	3
leiomyosarcoma	SK-UT-1	GX15-070 & PXD-101	0.1	24	48	1.220	1.215	1.211	1.215	0.162	3	0.092	1.28	3
leiomyosarcoma	SK-UT-1	GX15-070 & Vorinostat	0.02	24	48	1.227	1.208	1.196	1.210	0.182	3	0.066	1.14	3
leiomyosarcoma	SK-UT-1	MK-2206 & Doxorubicin	20	24	48	0.727	0.692	0.684	0.701	0.095	3	0.150	4.08	3
leiomyosarcoma	SK-UT-1	MK-2206 & Etoposide	1	24	48	0.710	0.771	0.832	0.771	0.051	3	0.153	3.87	3
leiomyosarcoma	SK-UT-1	MK-2206 & MK-8669	0.2	24	48	0.877	0.899	0.918	0.898	0.074	3	0.177	3.65	3
leiomyosarcoma	SK-UT-1	MK-8669 & Vorinostat	2	24	48	0.772	1.366	2.490	1.286	0.343	3	0.122	3.44	1
leiomyosarcoma	SK-UT-1	PXD-101 & Doxorubicin	0.33333	24	48	0.703	0.704	0.706	0.704		1	0.189	4.73	1
leiomyosarcoma	SK-UT-1	PXD-101 & Doxorubicin	1	24	48	0.493	0.492	0.491	0.492		1	0.264	5.23	1
leiomyosarcoma	SK-UT-1	PXD-101 & Etoposide	0.05	24	48	0.614	0.603	0.596	0.604	0.132	3	0.186	7.22	3
leiomyosarcoma	SK-UT-1	PXD-101 & Etoposide	3.33E-02	24	48	0.767	0.776	0.782	0.775		1	0.166	3.88	1
leiomyosarcoma	SK-UT-1	PXD-101 & Topotecan	0.33333	24	48	0.627	0.623	0.621	0.623		1	0.171	6.32	1
leiomyosarcoma	SK-UT-1	PXD-101 & Topotecan	2.5	24	48	0.459	0.448	0.441	0.449		1	0.177	5.17	1
leiomyosarcoma	SK-UT-1	Sorafenib & Topotecan	10	24	48	2.881	6.582	13.995	6.426		1	-0.063	3.73	1
leiomyosarcoma	SK-UT-1	Topotecan & Sorafenib	0.002	24	48	1.556	1.673	1.795	1.666	0.446	3	0.000	0.84	2
leiomyosarcoma	SK-UT-1	Vorinostat & Cisplatin	0.2	48	24	0.578	0.374	0.278	0.410	0.104	2	-0.004	0.94	2
leiomyosarcoma	SK-UT-1	Vorinostat & Etoposide	0.2	48	24	0.374	0.233	0.171	0.259	0.004	2	0.080	2.52	2
leiomyosarcoma	SK-UT-1	Vorinostat & MK-8669	0.05	24	48	0.851	0.863	0.887	0.867	0.033	4	0.117	2.86	2
leiomyosarcoma	SK-UT-1	Vorinostat & Topotecan	0.4	48	24	0.357	0.258	0.221	0.279	0.002	2	0.089	3.92	2
liposarcoma	SW-872	Ara-C & Dasatinib	0.25	24	48	0.264	0.282	0.294	0.280		1	0.194	2.95	1
liposarcoma	SW-872	Dasatinib & Ara-C	0.25	24	48	0.027	0.027	0.026	0.027		1	0.353	5.53	1
liposarcoma	SW-872	Dasatinib & Etoposide	0.4	48	14	0.254	0.229	0.213	0.232		1			
liposarcoma	SW-872	Dasatinib & Topotecan	0.66667	48	14	0.129	0.129	0.129	0.129		1			
liposarcoma	SW-872	Dasatinib & Topotecan	2.66667	48	14	0.229	0.141	0.102	0.158		1			
liposarcoma	SW-872	Dasatinib & Triciribine	1	24	48	0.538	0.536	0.535	0.536		1	0.180	7.76	1
liposarcoma	SW-872	GX15-070 & Dasatinib	0.01	24	48	1.244	1.231	1.222	1.232		1	0.093	1.71	1
liposarcoma	SW-872	GX15-070 & Dasatinib	0.02	24	48	1.115	1.104	1.097	1.106	0.004	2	0.101	2.29	2
liposarcoma	SW-872	GX15-070 & PXD-101	0.1	24	48	1.412	1.448	1.474	1.445	0.167	3	0.029	0.61	3
liposarcoma	SW-872	GX15-070 & Vorinostat	0.02	24	48	1.490	1.473	1.462	1.475		1	0.007	0.18	1
liposarcoma	SW-872	GX15-070 & Vorinostat	100	24	48	0.413	0.413	0.414	0.413		1	0.257	3.76	1
liposarcoma	SW-872	MK-2206 & Doxorubicin	20	24	48	0.807	0.728	0.688	0.741	0.134	3	0.136	3.01	3
liposarcoma	SW-872	MK-2206 & Etoposide	1	24	48	1.041	1.017	1.004	1.021	0.047	3	0.158	3.40	3
liposarcoma	SW-872	MK-2206 & MK-8669	0.2	24	48	1.128	1.209	1.278	1.205	0.073	3	0.188	3.47	3
liposarcoma	SW-872	MK-8669 & Vorinostat	2	24	48	1.817	3.830	6.411	3.547		1			
liposarcoma	SW-872	PXD-101 & Doxorubicin	0.33333	24	48	0.668	0.661	0.656	0.662		1	0.142	2.49	1
liposarcoma	SW-872	PXD-101 & Doxorubicin	1	24	48	1.165	1.164	1.164	1.164		1	0.068	4.75	1
liposarcoma	SW-872	PXD-101 & Etoposide	0.05	24	48	1.179	1.178	1.178	1.179	0.084	2	0.066	2.77	2
liposarcoma	SW-872	PXD-101 & Etoposide	3.33E-02	24	48	0.669	0.655	0.645	0.656		1	0.068	1.58	1
liposarcoma	SW-872	PXD-101 & Topotecan	0.33333	24	48	0.796	0.812	0.823	0.810		1	0.032	1.97	1
liposarcoma	SW-872	PXD-101 & Topotecan	2.5	24	48	1.037	1.041	1.044	1.041	0.057	2	0.018	1.13	2
liposarcoma	SW-872	Sorafenib & Topotecan	10	24	48	1.288	1.564	1.786	1.546	0.093	2	-0.027	1.40	1
liposarcoma	SW-872	Topotecan & Sorafenib	0.002	24	48	0.914	0.895	0.895	0.901	0.328	2	0.108	2.25	1
liposarcoma	SW-872	Triciribine & Dasatinib	1	24	48	0.203	0.198	0.195	0.198		1	0.302	10.62	1
liposarcoma	SW-872	Vorinostat & Cisplatin	0.2	48	24	0.663	0.605	0.569	0.613		1	0.088	3.43	1
liposarcoma	SW-872	Vorinostat & Etoposide	0.2	48	24	0.989	1.273	1.572	1.237	0.343	2	0.067	2.41	2
liposarcoma	SW-872	Vorinostat & MK-8669	0.05	24	48	1.051	0.953	0.895	0.966	0.139	3	0.057	2.21	2
fibrosarcoma	HT-1080	Ara-C & Dasatinib	0.25	24	48	0.482	0.402	0.356	0.414		1	0.251	5.62	1
fibrosarcoma	HT-1080	GX15-070 & Dasatinib	0.01	24	48	0.888	0.877	0.869	0.878		1	0.179	3.49	1
fibrosarcoma	HT-1080	GX15-070 & Dasatinib	0.02	24	48	0.652	0.638	0.630	0.640	0.109	3	0.244	3.86	3
fibrosarcoma	HT-1080	GX15-070 & Doxorubicin	1	24	48	0.775	0.778	0.780	0.777		1	0.109	3.64	1
fibrosarcoma	HT-1080	GX15-070 & PXD-101	0.1	24	48	0.972	0.990	1.002	0.988	0.180	3	0.132	1.61	3
fibrosarcoma	HT-1080	GX15-070 & Vorinostat	0.01	24	48	1.002	0.948	0.913	0.954		1	0.157	2.38	1
fibrosarcoma	HT-1080	GX15-070 & Vorinostat	0.02	24	48	0.858	0.869	0.877	0.868	0.138	3	0.135	1.62	3
fibrosarcoma	HT-1080	MK-8669 & Vorinostat	2	24	48	0.450	0.602	0.760	0.604	0.073	2	0.308	3.78	2
fibrosarcoma	HT-1080	PXD-101 & Doxorubicin	0.025	24	48	0.300	0.283	0.273	0.285		1	0.275	3.50	1

Sarcoma type	Cell line	Drug combination	Molar ratio	Time1	Time2	CI75	CI90	CI95	CI(mean)	CI(SEM)	n	EOHSA (mean)	neg log p-Value	n
fibrosarcoma	HT-1080	PXD-101 & Doxorubicin	1	24	48	0.676	0.678	0.679	0.677		1	0.266	5.02	1
fibrosarcoma	HT-1080	PXD-101 & Etoposide	0.025	24	48	0.753	0.754	0.755	0.754		1	0.153	3.39	1
fibrosarcoma	HT-1080	PXD-101 & Etoposide	0.05	24	48	0.688	0.691	0.692	0.690	0.021	2	0.182	4.34	2
fibrosarcoma	HT-1080	PXD-101 & Etoposide	3.33E-02	24	48	0.735	0.736	0.737	0.736		1	0.137	3.43	1
fibrosarcoma	HT-1080	PXD-101 & Topotecan	0.025	24	48	0.994	1.004	1.011	1.003		1	0.024	1.15	1
fibrosarcoma	HT-1080	PXD-101 & Topotecan	0.25	24	48	0.516	0.531	0.542	0.530		1	0.225	4.95	1
fibrosarcoma	HT-1080	PXD-101 & Topotecan	0.33333	24	48	0.609	0.610	0.610	0.610		1	0.138	2.83	1
fibrosarcoma	HT-1080	PXD-101 & Topotecan	2.5	24	48	0.609	0.609	0.609	0.609		1	0.156	3.59	1
fibrosarcoma	HT-1080	Selumetinib & Doxorubicin	1	24	48	0.899	0.924	0.942	0.922	0.079	2	0.127	3.01	2
fibrosarcoma	HT-1080	Selumetinib & Topotecan	1	24	48	1.105	1.211	1.289	1.202		1	0.044	0.29	1
fibrosarcoma	HT-1080	Topotecan & Sorafenib	0.002	24	48	0.997	0.862	0.782	0.880		1	0.037	0.76	1
fibrosarcoma	HT-1080	Vorinostat & Cisplatin	0.2	48	24	0.447	0.370	0.327	0.381		1	0.112	2.19	1
fibrosarcoma	HT-1080	Vorinostat & Etoposide	0.2	48	24	0.528	0.429	0.381	0.446	0.019	3	0.095	2.12	3
fibrosarcoma	HT-1080	Vorinostat & MK-8669	0.05	24	48	0.662	0.604	0.571	0.612	0.187	2	0.172	2.40	2
fibrosarcoma	HT-1080	Vorinostat & Topotecan	0.4	48	24	0.643	0.607	0.593	0.614	0.162	2	0.101	4.25	2

Supplemental Figure 1S.

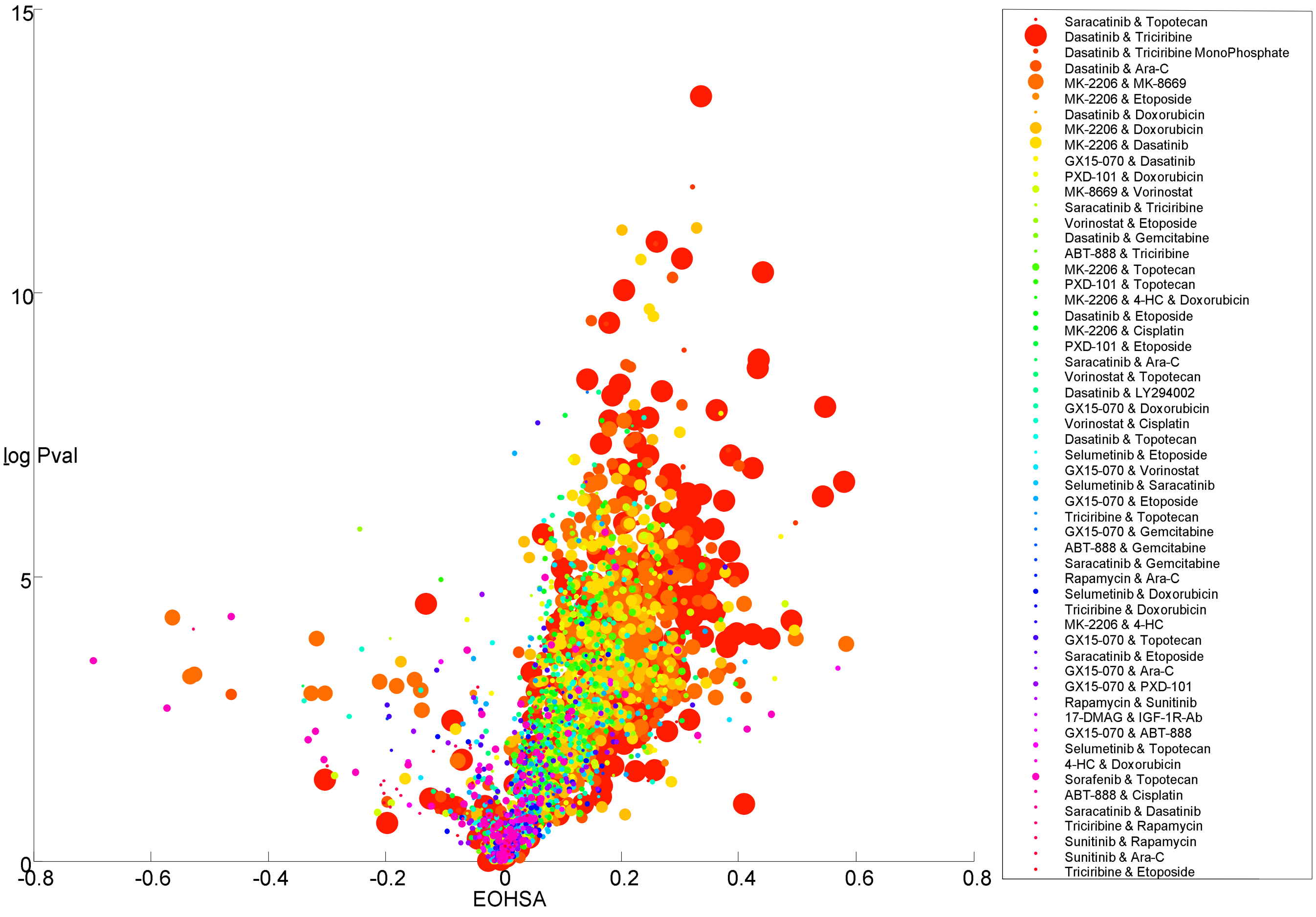

Supplement: Supplementary file 1 — Supplemental Figure 1S: Mean excess over highest single agent (EOHSA) vs. P value for tested drug combinations in 10 sarcoma cell lines. EOHSA was calculated from cell viability assay dose-response data for drug combinations and individual drugs for each combination. Results from combinations with potentially more significant synergy will show up in the upper right quadrant. Drug combinations are listed in legend in order of the mean EOHSA value with higher values appearing first. Symbol size represents the relative number of experiments included in the mean EOHSA value. Supplemental Table 1S: Concurrent 72 hour drug combination effect summary. The sarcoma cell line and molar drug ratio for each drug combination is indicated. Combination index (CI) values for effect levels of 0.75, 0.9 and 0.95 were calculated for each independent experiment by the method of Chou and Talalay and the mean CI value “CI(mean)” was calculated using the CI for each effect level. The standard error of the mean for CI values across independent experiments are shown “CI(SEM)”. EoHSA values and level of significance are represented in EoHSA (mean) and –log P-Value columns, respectively. Each line represents the calculated mean value for the number of independent experiments (n). Supplemental Table 2S: Sequential drug combination effect summary. The sarcoma cell line and molar drug ratio for each drug combination is indicated. Time1 indicates first drug incubation time (hours) for first drug listed in combination. Time2 indicates incubation time (hours) for secondary drug(s) added. Combination index (CI) values for effect levels of 0.75, 0.9 and 0.95 were calculated for each independent experiment by the method of Chou and Talalay and the mean CI value “CI(mean)” was calculated using the CI for each effect level. The standard error of the mean for CI values across independent experiments are shown “CI(SEM)”. EoHSA values and level of significance are represented in EoHSA(mean) and –log P-Valu [file 365723.f1.pdf]
